# The PACS-2 protein and trafficking motifs in CCHFV Gn and Gc cytoplasmic domains govern CCHFV assembly

**DOI:** 10.1080/22221751.2024.2348508

**Published:** 2024-04-25

**Authors:** Anupriya Gautam, Alexandre Lalande, Maureen Ritter, Natalia Freitas, Solène Lerolle, Lola Canus, Fouzia Amirache, Vincent Lotteau, Vincent Legros, François-Loïc Cosset, Cyrille Mathieu, Bertrand Boson

**Affiliations:** a CIRI – Centre International de Recherche en Infectiologie, Univ. Lyon, Université Claude Bernard Lyon 1, Lyon, France; b Laboratory P4-Jean Mérieux, Lyon, France; c Campus vétérinaire de Lyon, VetAgro Sup, Université de Lyon, Marcy-l’Etoile, France

**Keywords:** CCHFV, assembly, trafficking, glycoprotein, nucleoprotein

## Abstract

The Crimean-Congo hemorrhagic fever virus (CCHFV) is a tick-borne bunyavirus that causes high mortality in humans. This enveloped virus harbors two surface glycoproteins (GP), Gn and Gc, that are released by processing of a glycoprotein precursor complex whose maturation takes place in the ER and is completed through the secretion pathway. Here, we characterized the trafficking network exploited by CCHFV GPs during viral assembly, envelopment, and/or egress. We identified membrane trafficking motifs in the cytoplasmic domains (CD) of CCHFV GPs and addressed how they impact these late stages of the viral life cycle using infection and biochemical assays, and confocal microscopy in virus-producing cells. We found that several of the identified CD motifs modulate GP transport through the retrograde trafficking network, impacting envelopment and secretion of infectious particles. Finally, we identified PACS-2 as a crucial host factor contributing to CCHFV GPs trafficking required for assembly and release of viral particles.

## Introduction

The Crimean-Congo hemorrhagic fever virus (CCHFV) belongs to the genus *Orthonairovirus* under the *Nairoviridae* family and the *Bunyavirales* order. CCHFV was first associated with the manifestation of febrile disease in Crimea in 1944 [1] and in Congo in 1956 [2], and can cause fatality rates that surpass 30%. It is now prevalent in various regions, including the Middle East, Southeast Asia, Africa, and Southern and Eastern Europe [3–5]. Ticks of the genus *Hyalomma* have been identified as the primary vector and reservoir of CCHFV, though other tick species may potentially serve as hosts for the virus in regions where it is endemic [6]. Reports have shown that CCHFV has the potential to infect various animal species, most notably livestock, without causing apparent illness. These animals facilitate the transmission of CCHFV from infected ticks to uninfected ticks through co-feeding or by feeding on viremic animals. The primary modes of transmission of CCHFV to humans are tick bites and the handling of infected livestock [7]. Subsequently, CCHFV propagates throughout the body and target different organs, including the liver, where it induces multiple lesions, failures, and vascular dysfunction.

**Figure 1.**
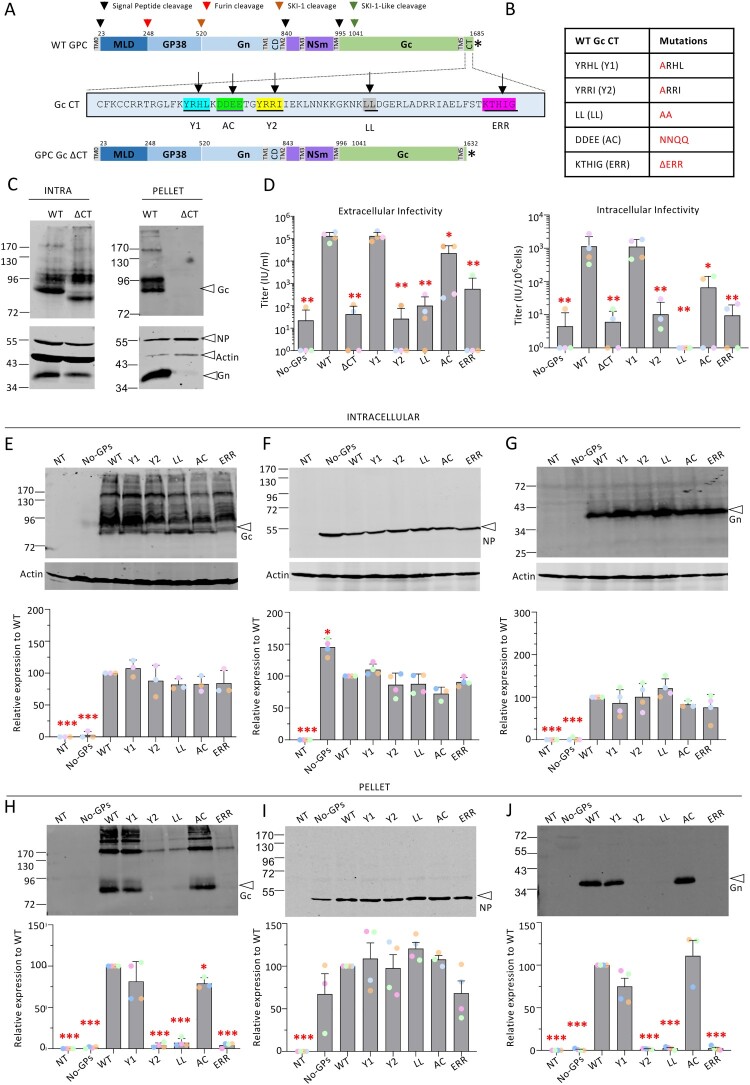
**Infectivity and viral incorporation of CCHFV Gc cytoplasmic tail mutants.** (**A**) Schematic representation of the GPC polyprotein encoded by CCHFV wt-M cDNA (WT GPC) and mutant GPC harbouring deletion of Gc cytoplasmic tail (GPC Gc ΔCT). The polyprotein precursor organization and positions of the first amino-acid residues after cleavage marking protein boundaries (MLD, GP38, Gn, NSm, and Gc) within the CCHFV polyprotein are shown. The stars indicate the position of the stop codon. The N-terminal signal peptide (TM0) and putative transmembrane domains (TM1 to TM5) are shown as grey boxes, signal peptidase cleavage sites are indicated by black arrows and other host protein convertase cleavage sites are indicated by red, orange and green arrows. The bottom part shows the cytoplasmic tail (CT) sequence of Gc. Several trafficking motifs were identified, underlined and boxed in different colours: two tyrosine-based motifs, Y1 and Y2 (blue and yellow); di-leucine motif, LL (grey); acidic cluster, AC, (green); endoplasmic reticulum retrieval domain, ERR (pink). Deletion of the putative trafficking motifs in the cytoplasmic tail of Gc is indicated as ΔCT. (**B**) The mutations introduced in the above Gc motifs are shown in the Table. (**C**) Intracellular levels and processing (INTRA) and viral incorporation (PELLET) of CCHFV wild type glycoproteins compared to Gc-ΔCT GPC mutant. Lysates of Huh7.5 cells producing tc-VLPs generated with WT GPC *vs*. Gc-ΔCT GPC mutant were analysed by Western blotting with antibodies against the indicated proteins including Gn, Gc, NP and cellular actin. Cell supernatants containing tc-VLPs were concentrated by ultracentrifugation through 20% sucrose cushions, resuspended in Opti-MEM medium and analysed by Western blot. (**D**) Infectivity titres of intracellular and extracellular CCHFV tc-VLPs bearing mutant Gc CT proteins. At 72 h post-transfection, clarified supernatants and cell-associated tc-VLPs were used to infect Huh7.5 cells pre-transfected with L and N expression vectors, and titres were determined by FACS analysis at 24 h post-infection. (**E-G**) Intracellular levels and processing of CCHFV WT GPs compared to Gc CT mutant proteins. Representative Western blot analysis and relative quantification of intracellular Gc, NP and Gn compared to WT proteins (lower panels). Protein band intensities were quantified and normalized relative to actin and expressed as fold change compared to WT (lower panels). (**H-J**) tc-VLP containing cell supernatants were pelleted by ultracentrifugation through 20% sucrose cushions, resuspended in Opti-MEM medium and analysed by Western blot. Representative Western blot analysis and relative quantification of Gc, NP and Gn expressed as fold change compared to WT proteins (lower panels). Molecular weight markers are marked to the left (kDa). Statistical significance was determined using parametric student-t test compared with WT proteins. Average number of repeats for intracellular CCHFV proteins: Gn = 4, Gc = 3, NP = 4. Average number of repeats for CCHFV proteins in pellets: Gn = 4, Gc = 4, NP = 4. Average number of repeats for extracellular and intracellular infectivity assays: n = 4. The values are displayed as means ± SEM. Each dot in the graphs corresponds to the value of an individual experiment and dots from one colour are from the same experiment.

CCHFV is an enveloped virus with a tri-segmented negative – or ambisense-strand RNA genome associated with virus-encoded nucleoproteins (NP) and an RNA-dependent RNA polymerase enclosed within a host-derived lipid bilayer, at the surface of which two surface glycoproteins (GPs) – Gn and Gc heterodimer – are inserted. The three genomic RNA segments are the large (L) segment, which encodes the polymerase, the small (S) segment, which encodes NP and a non-structural protein (NSs), and the medium (M) segment, which encodes a single polyprotein precursor (Figure 1A), namely the glycoprotein precursor complex (GPC). The two surface GPs are produced by several cleavage events on GPC that also give rise to several nonstructural proteins [8,9]. Viral assembly utilizes membrane-associating determinants, featuring a leader N-terminal signal peptide (SP), two internal SPs and five transmembrane domains (TMD), two intermediate GP precursors (preGn (140 kDa) and preGc (85 kDa)), a double-membrane-spanning non-structural protein called NSm (a mucin-like protein (MLD) containing a large number of predicted O-glycosylation sites), and three secreted proteins of poorly-known functions: GP38, GP85, and GP160 (Figure 1A). PreGn is converted to Gn following the cleavage by SKI-1/S1P protease in ER-*cis* Golgi compartments, while preGc is cleaved by an unknown SKI-1-like protease to generate Gc [10]. Like for related hemorrhagic viruses such as the Severe fever with thrombocytopenia syndrome virus (SFTSV) and Bunyamwera virus (BUNV), which are from the *Bandavirus* and *Orthobunyavirus* genera (*Bunyavirales* order), respectively, the Gn/Gc GPs form multimers, suggesting that CCHFV GPs may form a complex in order to stabilize the fusion domains in the viral membrane proximal region during assembly and egress of CCHFV [11,12]. However, the site where CCHFV particles are assembled and enveloped remains undefined. Previous work on *Bunyavirales* viruses suggest that assembly occurs on the Golgi membranes [13]. Since virus GPs are synthesized in the endoplasmic reticulum (ER), a key question is about how CCHFV GPs are transported from the ER to the virion assembly site, which remains to be determined.

Protein traffic through the secretory pathway requires tight regulations and highly synchronized interactions of several cellular factors [14]. The secretory pathway is subdivided into two membrane populations: the ER/Golgi system, which is needed for oligomerization, folding, and co-posttranslational modifications of proteins that shuttle along the secretory pathways [15,16], and the *trans*-Golgi network (TGN)/endosomal system, which is important for sorting, export, and recovery of various soluble and membrane-associated secretory proteins.

Several types of trafficking motifs are typically contained in the cytoplasmic tails (CT) or cytoplasmic domain (CD) of viral surface glycoproteins [17–19], which may also be the case for bunyaviruses [20,21], and regulate various stages of GPs, from their biogenesis to their ultimate intracellular localization. As for CCHFV, the Gn GP contains an ectodomain of 176 residues that is followed by two TMDs with, in between, a particularly long CD of 94 residues [8,10,22], which has been shown by NMR analysis to possess a unique dual CCHC-type zinc finger (ZF) fold that is capable of binding viral RNA [23]. On the other hand, the Gc glycoprotein contains an ectodomain of 481 residues [24] that is followed by a single TMD followed by a 63 residue-long CT of unknown function [25].

Several host factors can be coopted by enveloped viruses to mediate intracellular trafficking of their GPs. For example, the adaptor proteins (AP) AP-1, AP-2 and AP-3 [26] mediate different sorting events, such as internalization from plasma membrane and sorting to endosomes [27], and the COPI coatomer mediates retrograde transport of cargos from TGN to ER [28] whereas two proteins of the PACS family, PACS-1 and PACS-2, mediate retrograde transport from endosomal pathway to TGN and control the ER localization [29], respectively, through interactions with AP-1 and AP-3 (PACS-1) and COPI (PACS-2).

How CCHFV exploits these cellular trafficking networks and cellular host components during all stages of virus assembly and/or egress remains poorly defined. Here, using both a CCHFV minigenome-reporter transcription and entry-competent virus-like particle (tc-VLP) assay [30], mimicking viral particles [31], and full length, live virus, we aimed at understanding how CCHFV GPs make use of host factors and pathways to target the site of assembly and promote envelopment of its viral particles. Through a strategy allying functional assays with biochemical and intracellular imaging analyses, we investigated the role of putative membrane trafficking motifs that we identified in the CDs of CCHFV GPs, how their mutagenesis could impact envelopment and production of infectious viral particles, and what are some cellular factors involved in the above. Altogether, our results indicate that several CCHFV GP CD motifs have specific functions to transport Gn and Gc GPs through various parts of the membrane trafficking network to the virion assembly site and that the PACS-2 connector host protein plays a crucial role in the above.

## Results

**Gc cytoplasmic determinants are essential for the formation of infectious particles.** Using consensus sequences that were determined elsewhere, such as tyrosine-based motifs (YXXΦ [Φ standing for an amino acid with a bulky hydrophobic side chain and X for any amino acid]) [32,33], dileucine motifs (DXXLL and [DE]XXXL[LI]) [26,33], acidic cluster motifs [33,34], and di-lysine motifs [28,35], we identified several putative membrane trafficking motifs in the cytoplasmic tail (CT) of CCHFV Gc glycoprotein (Figure 1A): two tyrosine-based motifs (Y1 and Y2), an acidic cluster motif (AC), a di-leucine motif (LL), and an ER retrieval motif (ERR). We noticed from multiple sequence alignment analysis that these motifs are highly conserved among various CCHFV isolates (Supplementary Fig. 1A, 1C). These potential determinants were mutated (Figure 1B) in the full context of GPC expression vectors to raise mutant Gc proteins whose properties were investigated using a CCHFV transcriptionally – and entry-competent virus-like particle (tc-VLP) production and infection assay [30,36].

We evaluated the role of these putative trafficking motifs on virion assembly, GP incorporation, and infectivity in Huh7.5 hepatoma cells, as the liver is one of the important CCHFV target organs. We first generated a mutant GPC harbouring a deletion of most Gc CT (Gc-ΔCT) that was used to produce tc-VLPs by co-transfection of Huh7.5 cells with a CCHFV minigenome encoding GFP, CCHFV RNA polymerase (L) and nucleoprotein (NP) expression constructs together with constructs expressing either wild type (WT) GPC or Gc-ΔCT GPC (Figure 1C). Whole-cell lysates of tc-VLP producer cells (defined as "Intracellular” in all figures) and the corresponding supernatants purified by ultracentrifugation through a sucrose cushion (defined as “Pellet” in the Figures) were analysed by SDS-PAGE and Western blot at 72 h post-transfection (Figure 1C) to assess incorporation of mutant GPs on secreted viral particles. NP intracellular expression and secretion in cell supernatants were not significantly different between WT and Gc-ΔCT tc-VLPs (Figure 1C). In agreement with previous studies [10,30,31,36], the expression of WT GPC raised mature Gn protein at 37 kDa (Figure 1C) and Gc protein as a preGc precursor of 85 kDa that was converted to mature Gc (75 kDa). In contrast with WT GPC, the Gc-ΔCT GPC raised lower preGc and Gc bands due to the deletion of Gc cytoplasmic tail. Western blot analyses of pellets of tc-VLP producer cell supernatants indicated that while Gn and Gc were readily incorporated into particles for WT GPC, tc-VLPs generated with the Gc-ΔCT mutant displayed no or poor Gc and Gn levels, suggesting a defect in virion incorporation of either GP.

We examined the ability of this Gc-ΔCT GPC mutant to support the formation and release of extracellular and intracellular infectious CCHFV tc-VLPs. We used tc-VLPs generated in the absence of GPC, which does not generate infectious particles (No-GPs in Figure 1D) to set up the thresholds of infectivity assessments. In agreement with these above results, the deletion of the CT of Gc resulted in a complete loss of both extracellular and intracellular infectivity (Figure 1D), underscoring the presence of critical determinants in Gc CT that allow envelopment and production of infectious viral particles.

Next, we examined the infectivity of the GPC mutants generated in the identified Gc CT motifs (Figure 1A, B). We found that while the Y1 mutant allowed the formation and release of infectious tc-VLPs at levels identical to WT tc-VLPs, the other CT mutants yielded lower (AC mutant) or hardly detectable (Y2, LL, ERR mutants) infectivity for both intracellular and extracellular tc-VLPs. Collectively, these results indicate that several Gc CT determinants play important roles in the assembly and release of infectious tc-VLPs.

Then, to further understand the functions of Gc CT motifs, we analysed the effect of their mutation on Gn and Gc expression and processing, NP expression, and secretion of particle-associated viral proteins (Figure 1E–J). Expression of WT and mutant Gc GPC raised similar intracellular levels of NP, Gc and Gn expression (Figure 1E–G). Yet, in contrast to Y1 and AC Gc mutant GPCs whose Gn and Gc GPs were not or only slightly less well incorporated into particles compared to WT GPC, the other Gc CT mutant GPC constructs did not allow secretion of tc-VLPs displaying Gc or Gn GPs (Figure 1H–J), which fully supported the results of infection assays (Figure 1D).

Altogether, these results indicated that the motifs identified in Gc CT regulate an early stage of virion production prior to secretion.

**Gc CT determinants play a role in cellular trafficking of CCHFV Gc glycoprotein.** The processing and maturation steps of WT GPC are initiated in the ER and are followed by PreGn and PreGc transport through the secretion pathway and ultimately to the proposed site of CCHFV assembly and envelopment, presumably near the Golgi complex [13]. Tyrosine-based motifs, di-leucine motifs, acidic clusters and ER retrieval motifs are involved in protein retrograde trafficking from plasma membrane to endosomes, from endosomes to Golgi and from Golgi to ER. Thus, we assessed the role of Gc CT determinants on Gc intracellular trafficking and localization by immunofluorescence (IF) studies using cellular markers specific for the Golgi (GM130), early (Rab5) and late (Rab7) endosomes.

**Figure 2.**
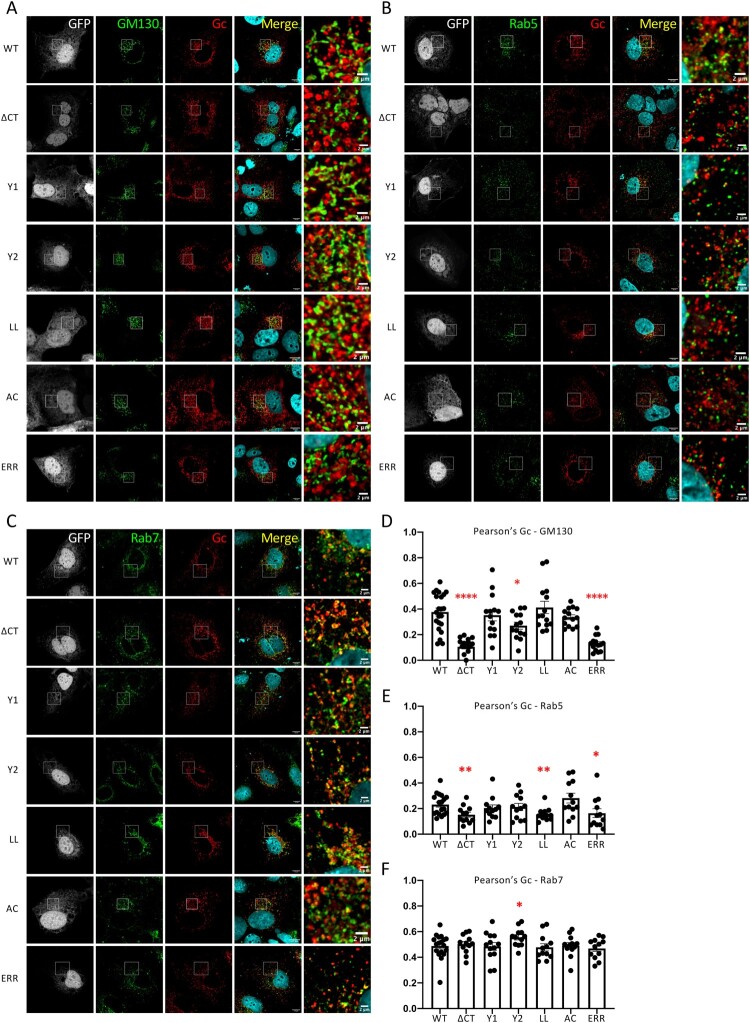
**Intracellular localization of CCHFV Gc cytoplasmic tail determinants**. (**A, D**) Golgi localization of CCHFV Gc glycoprotein. Confocal microscopy analysis of Huh7.5 cells producing tc-VLPs encoding a GFP marker that were generated with WT GPC *vs*. GPC harbouring Gc cytoplasmic tail mutants (ΔCT, Y1, Y2, LL, AC, ERR). At 48 h post-transfection, cells were fixed, permeabilized with Triton X-100, and stained for GFP (grey channel), Golgi (anti-GM130, green channel), Gc (11E7, red channel), and nuclei (Hoechst, blue channel). (**B, E**) Early endosome localization of CCHFV Gc glycoprotein. As described in (A), cells were fixed, permeabilized with Triton X-100, and stained for GFP (grey channel), Rab5 (anti-Rab5, green channel), Gc (11E7, red channel), and nuclei (Hoechst, blue channel). (**C, F**) Late endosome localization of CCHFV Gc glycoprotein. As described in (A), cells were fixed and stained for GFP (grey channel), Rab7 (anti-Rab7, green channel), Gc (11E7, red channel), and nuclei (Hoechst, blue channel). Scale bars represent 10 μm, zooms from squared area represent 2μm. Pearson’s coefficients were calculated using FIJI (JACoP) on 5 cells from 3 separated experiments (15 cells in total) and expressed as means ± SEM. Statistical significance was determined using non-parametric two-tailed Mann-Whitney test. The values are displayed as means ± SEM. Each dot in the graphs corresponds to the value of an individual cell.

As reported before [36], Gc expressed from WT GPC is localized in Huh7.5 cells throughout the secretory pathway, without significant accumulation in Golgi and late endosomes (Figure 2). Yet, in contrast to WT GP, we found that the Gc-ΔCT mutant had impaired co-localization with GM130 (Figure 2A, D) and Rab5 markers (Figure 2B, E), likely owing to the removal of intracellular trafficking motifs in Gc CT. Moreover, we found that the ΔCT mutant displayed increased detection of Gc at the cell surface as shown by IF in non-permeabilized cells (Supplementary Fig. 2A), suggesting that this mutant accumulated at the plasma membrane (PM).

Altogether, these results underscored the role of CT motifs that allow Gc trafficking from the PM to intracellular organelles or prevent Gc exposition at the PM.

We therefore sought to address the individual contribution of the putative trafficking motifs present in Gc CT to Gc intracellular traffic and localization, and to correlate this with assembly and release of viral particles.

First, we investigated the levels of cell surface expression of the mutants by IF in non-permeabilized cells. We found that compared to WT GPC and other mutant GPCs, the Gc Y1 mutant displayed higher levels of expression at the surface (Supplementary Fig. 2A). This suggested that mutation of the Y1 motif could prevent Gc internalization, which induced its cell surface accumulation, and agreed with higher cell–cell fusion levels induced by this mutant as compared to WT GPC and other Gc CT motif mutant GPC (Supplementary Fig. 2B). Importantly, this result argues that Gc’s CT contains motifs that allow its trafficking from the PM to intracellular organelles rather than preventing its cell surface exposition.

Second, when we addressed the above determinants in Gc CT (Figure 1A, B), we found that the Gc AC mutant and, with statistical significance, the Y2 and ERR mutants exhibited reduced colocalization with GM130 marker, underscoring impaired trafficking to or within the Golgi (Figure 2A, D). Then, when we investigated Gc localization in early endosomes, we found that the Gc LL and ERR mutants exhibited a decreased co-localization with Rab5 marker, as compared to WT GPC, indicating that retrograde trafficking from early endosomes to Golgi may be impaired for these mutants (Figure 2B, E). Yet, in contrast with WT Gc, the Gc AC mutant showed slightly increased co-localization with Rab5 (Figure 2B, E). This observation suggested an impaired retrograde trafficking of the Gc AC mutant from the early endosomes to the Golgi apparatus. From early endosomal compartments, a subset of proteins can be targeted to late endosomes [37]. We thus addressed the co-localization of Gc mutants with the late endosomal marker, Rab7. In contrast to Gc expressed from WT GPC, the Y2 mutant showed increased co-localization with Rab7 (Figure 2C, F), suggesting that the mutation of its tyrosine-based motif impairs its trafficking from the late endosomes to the upstream compartment, possibly to lysosomes or lysosome-related organelles [33].

Altogether, these results indicated that in the context of GPC, several determinants of the CT domain of Gc cooperate to allow intracellular trafficking of CCHFV Gc GP from the PM to the virion assembly site.

**Figure 3.**
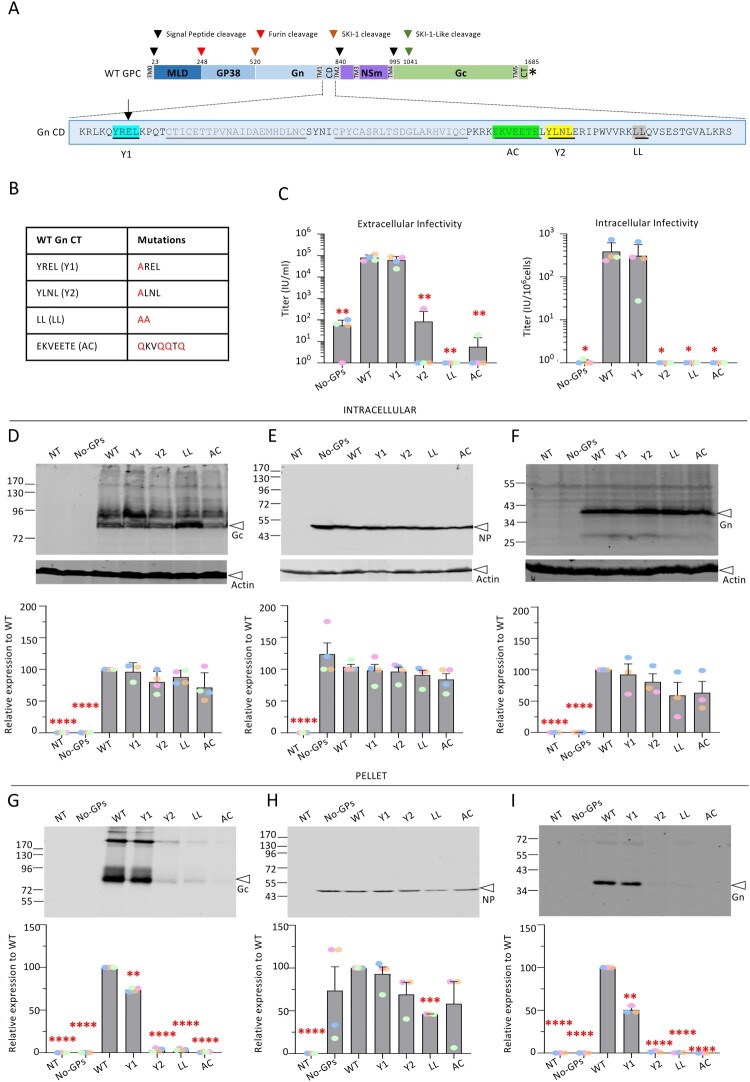
**Infectivity and viral incorporation of CCHFV Gn cytoplasmic tail mutants.** (**A**) Schematic representation of the GPC polyprotein encoded by CCHFV wt-M cDNA (WT GPC). The polyprotein precursor organization and positions of the first amino-acid residues after cleavage marking protein boundaries (MLD, GP38, Gn, NSm, and Gc) within the CCHFV polyprotein are shown. The star indicates the position of the stop codon. The N-terminal signal peptide (TM0) and putative transmembrane domains (TM1 to TM5) are shown as grey boxes, signal peptidase cleavage sites are indicated by black arrows and other host protein convertase cleavage sites are indicated by red, orange and green arrows. The bottom part shows the cytoplasmic domain (CD) of Gn. Several trafficking motifs were identified, underlined and boxed in different colours: two tyrosine-based motifs, Y1 and Y2 (blue and yellow); di-leucine motif, LL (grey); acidic cluster, AC (green). (**B**) The mutations introduced in the above Gn motifs are shown in the Table. (**C**) Infectivity titres of intracellular and extracellular CCHFV tc-VLPs bearing mutant Gn CD proteins. At 72 h post-transfection, clarified supernatants and cell-associated tc-VLPs were used to infect Huh7.5 cells pre-transfected with L and N expression vectors, and titres were determined by FACS analysis at 24 h post-infection. (**D-F**) Intracellular levels and processing of CCHFV WT GPs compared to Gn CD mutant proteins. Representative Western blot analysis and relative quantification of intracellular Gc, NP and Gn expressed compared to WT proteins (lower panels). Protein band intensities were quantified and normalized relative to actin and expressed as fold change compared to WT (lower panels). (**G-I**) tc-VLP containing cell supernatants were pelleted by ultracentrifugation through 20% sucrose cushions, resuspended in Opti-MEM medium and analysed by Western blot. Representative Western blot analysis and relative quantification of Gc, NP and Gn expressed as fold change compared to WT proteins (lower panels). Molecular weight markers are marked to the left (kDa). Statistical significance was determined using parametric student-t test compared with WT proteins. Average number of repeats for intracellular CCHFV proteins: Gn = 3, Gc = 4, NP = 4. Average number of repeats for CCHFV proteins in pellets: Gn = 3, Gc = 4, NP = 4. Average number of repeats for extracellular and intracellular infectivity assays: n = 4. The values are displayed as means ± SEM. Each dot in the graphs corresponds to the value of an individual experiment and dots from one colour are from the same experiment.

**Mutations in Gn cytosolic determinants impact the formation of CCHFV infectious particles.** We identified several putative membrane trafficking motifs in the CD of CCHFV Gn glycoprotein (Figure 3A). Similar to Gc CT, the multiple sequence alignment analysis of Gn CD also revealed highly conserved motifs (Supplementary Fig. 1B, 1C): two tyrosine-based motifs (Y1 and Y2), an acidic cluster motif (AC) and a di-leucine motif (LL). These potential determinants were mutated in the context of the GPC expression vector to raise mutant Gn proteins upon CCHFV tc-VLP production (Figure 3B).

We found that while the mutation of the Y1 motif allowed the formation and release of infectious tc-VLPs at levels identical to WT tc-VLPs, the other CD mutants (Y2, LL, and AC mutants) yielded strongly reduced or no infectivity for both intracellular and extracellular tc-VLPs (Figure 3C). These results indicated that the latter determinants play essential roles in GP incorporation and release of infectious virions.

Then, to better understand the functions of Gn CD motifs, we analysed the effect of their mutation on Gn and Gc expression and processing, NP expression and secretion of particle-associated viral proteins (Figure 3D–I). Expression of WT and mutant GPC raised similar intracellular levels of NP, Gc and Gn expression (Figure 3D–F). Yet, in sharp contrast to Gn Y1 mutant whose Gn and Gc GPs were only slightly less well incorporated into viral particles, as compared with WT GPs, the other Gn CD mutant constructs raised tc-VLPs that had no detectable Gn and/or Gc GPs (Figure 3G–I), which fully supported the results of infection assays (Figure 3C). Further, we noticed that di-leucine mutation in Gn led to less NP secretion in pellet fraction (Figure 3H).

Altogether, these results indicated that motifs identified in Gn cytosolic domain regulate an early stage of assembly before virion secretion.

To further investigate the function of these Gn determinants in assembly of viral particles, we analysed tc-VLP-producer cells by confocal microscopy using antibodies against Gc and intracellular markers. However, due to the lack of Gn antibodies suitable for IF assays, we could not perform the analysis of Gn intracellular localization.

**Figure 4.**
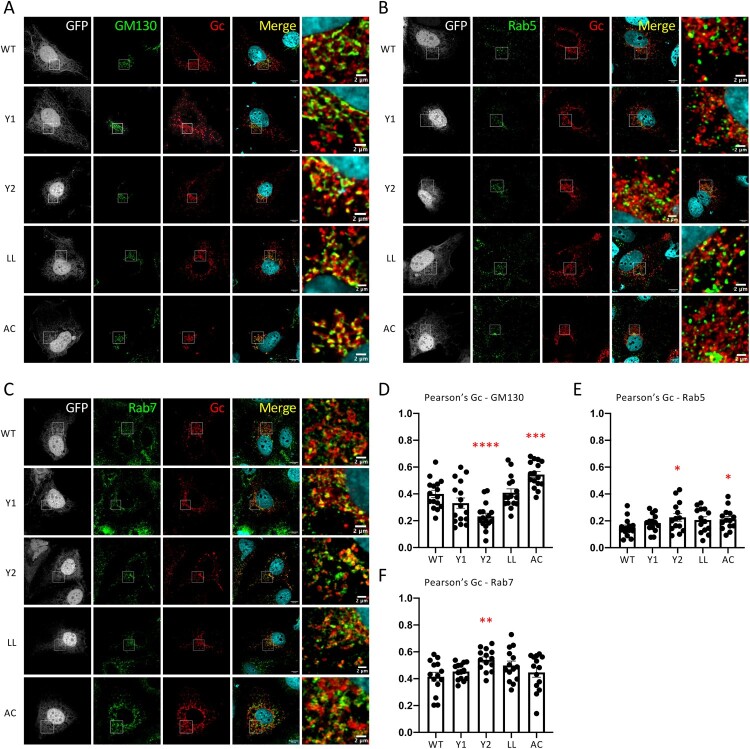
**Intracellular localization of CCHFV Gn cytoplasmic tail mutants.** (**A, D**) Golgi localization of CCHFV Gc glycoprotein. Confocal microscopy analysis of Huh7.5 cells producing tc-VLPs encoding a GFP marker that were generated with WT GPC *vs*. GPC harbouring Gn cytoplasmic domain mutants (Y1, Y2, LL, AC). At 48 h post-transfection, cells were fixed, permeabilized with Triton X-100, and stained for GFP (grey channel), Golgi (anti-GM130, green channel), Gc (11E7, red channel), and nuclei (Hoechst, blue channel). (**B, E**) Early endosome localization of Gc glycoprotein. As described in (A), cells were fixed, permeabilized with Triton X-100, and stained for GFP (grey channel), Rab5 (anti-Rab5, green channel), Gc (11E7, red channel), and nuclei (Hoechst, blue channel). (**C, F**) Late endosome localization of Gc glycoprotein. As described in (A), cells were fixed and stained for GFP (grey channel), Rab7 (anti-Rab7, green channel), Gc (11E7, red channel), and nuclei (Hoechst, blue channel). Scale bars represent 10 μm, zooms from squared area represent 2μm. Pearson’s coefficients were calculated using FIJI (JACoP) on 5 cells from 3 separated experiments (15 cells in total) and expressed as means ± SEM. Statistical significance was determined using non-parametric two-tailed Mann-Whitney test. The values are displayed as means ± SEM. Each dot in the graphs corresponds to the value of an individual cell.

Like for the Gc Y2 mutant (Figure 2), we found that the Gn Y2 mutant induced a defect in the distribution pattern of Gc in the Golgi (Figure 4A, D), suggesting that tyrosine domains of both Gn and Gc work in a similar way to promote CCHFV GP intracellular localization where mutations in Gn or Gc can impact traffic of the other protein [38] as they likely form a heterodimer [11,12]. Surprisingly, we also found that the Gn AC mutant induced a stronger Gc co-localization with GM130, indicating that the GP can reach the Golgi but displays impaired trafficking through the Golgi.

Finally, when we analysed Gc co-localization with early endosomes (Rab5 marker), we found that Gn Y2 and AC mutants showed a slight increase in Gc co-localization with early endosomes (Figure 4B, E). Yet, we noticed that in contrast to WT GPC, the Gn Y2 mutant exhibited increased Gc co-localization with the Rab7 late endosomal marker (Figure 4C, F).

Altogether, these results indicated that mutations of Gn CD motifs which induce GP accumulation in various cellular organelles may block retrograde transport to the trans-Golgi and prevent GP incorporation on viral particles.

**Unlike PACS-1, the PACS-2 adaptor is a critical factor controlling virion incorporation and secretion of CCHFV glycoproteins**. Our results revealed that the AC motif in Gn is important to promote CCHFV GP intracellular trafficking and/or incorporation on viral particles since its mutation induced accumulation of Gc in early endosomes and Golgi, and prevented GP assembly and release of infectious particles (Figures 3 and 4). Since some Golgi resident transmembrane proteins have been shown to use AP-1 mediated retrograde transport from endosomes to trans-Golgi network (TGN) through binding of the cellular adaptor PACS-1 [29] to their acidic cluster, we investigated whether PACS-1 down-regulation could impair CCHFV particles assembly.

**Figure 5.**
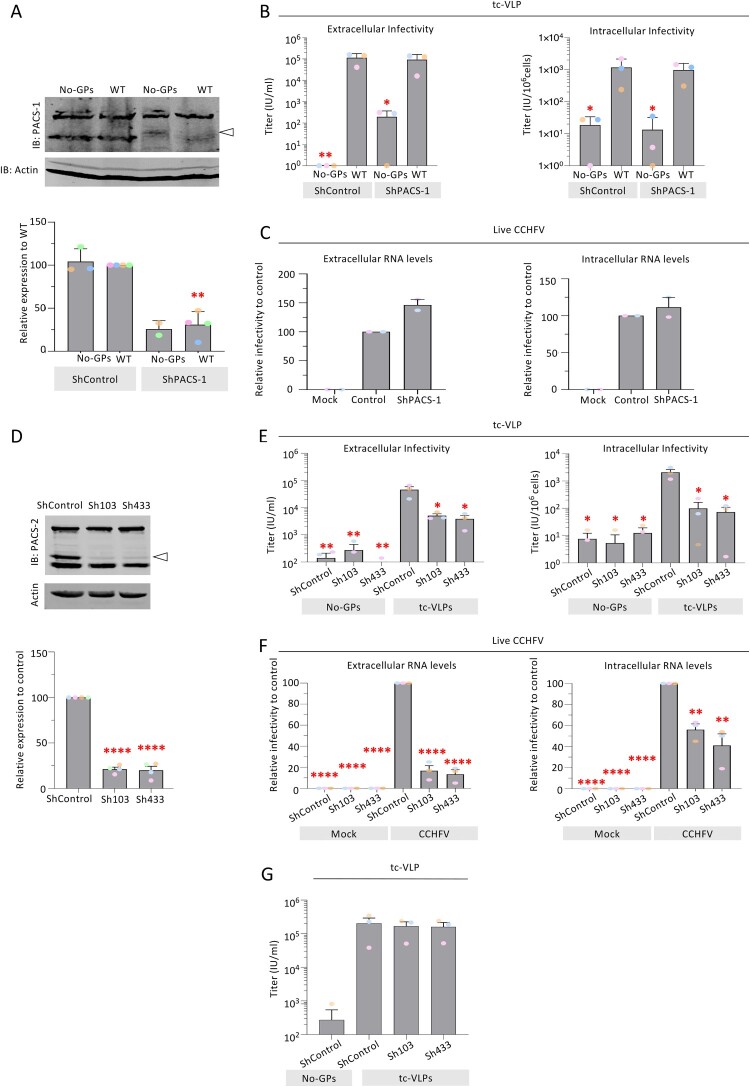
**Production of infectious CCHFV particles from PACS-1 or PACS-2 knockdown cells.** Downregulation of PACS-1 or PACS-2 was achieved in Huh7.5 cells *via* lentiviral vectors expressing PACS-1 (ShPACS-1), PACS-2 (Sh103, Sh433) or control shRNA (ShControl). The cells were then used to produce CCHFV tc-VLPs harbouring WT GPC or no GPC (No-GPs). (**A**) Representative Western blot analysis and relative quantification of PACS-1 expressed as fold change compared to PACS-1 levels expressed in cells transduced with a control shRNA (ShControl). (**B**) Infectivity titres of CCHFV tc-VLPs produced in the presence *vs*. in the absence of PACS-1 shRNA. At 72 h post-transfection, clarified supernatants and cell-associated tc-VLPs were used to infect Huh7.5 cells pre-transfected with L and N expression vectors, and titres were determined by FACS analysis at 24 h post-infection. (**C**) Viral characteristics of full length, live CCHFV produced in PACS-1 KD or control cells. The cells were infected with live CCHFV at an MOI of 0.01. Supernatants (extracellular) and cells (intracellular) were harvested at 24 h post infection. Viral RNA in the supernatants and cells were determined using RTqPCR. (**D**) Representative Western blot analysis and relative quantification of PACS-2 expressed as fold change compared to PACS-2 levels expressed in cells transduced with a control shRNA. (**E**) Infectivity titres of CCHFV tc-VLPs produced in the presence *vs*. in the absence of PACS-2 shRNA. At 72 h post-transfection, clarified supernatants and cell-associated tc-VLPs were used to infect Huh7.5 cells pre-transfected with L and N expression vectors, and titres were determined by FACS analysis at 24 h post-infection. (**F**) Viral characteristics of CCHFV produced in PACS-2 KD or control cells. The cells were infected with live CCHFV at an MOI of 0.01. Supernatants (extracellular) and cells (intracellular) were harvested at 24 h post infection. Viral RNA in the supernatants and cells were determined using RTqPCR. (**G**) Infectivity of tc-VLPs in PACS-2 down-regulated cells. Huh7.5 cells down-regulated for PACS-2 (Sh103, Sh433) or control shRNA (ShControl) were pre-transfected with L and N expression vectors. Twenty-four hours later, cells were infected with CCHFV tc-VLPs harbouring WT GPC or no GPC (No-GPs) and titres were determined by FACS analysis at 24 h post-infection. Average number of repeats for extracellular and intracellular infectivity assays: n = 3. The values are displayed as means ± SEM. Each dot in the graphs corresponds to the value of an individual experiment and dots from one colour are from the same experiment.

We downregulated PACS-1 through expression of a previously validated [39] short hairpin RNA (ShPACS-1) in producer cells. While we achieved a knockdown efficiency of PACS-1 of up to 80% in Huh7.5 cells (Figure 5A), this did not influence the production of infectious viral particles, as shown for both tc-VLP (Figure 5B) and full length, live CCHFV particles (Figure 5C) produced in PACS-1 downregulated Huh7.5 cells. Hence, we concluded that PACS-1 is not a crucial host factor for the transportation of CCHFV glycoproteins to the assembly site.

Next, we investigated the alternative possibility that the PACS-2 protein, which also binds acidic cluster motifs [40], could regulate intracellular trafficking of CCHFV GPs. We expressed in tc-VLP producer cells two distinct short hairpin RNAs (Sh103 and Sh433), which readily induced PACS-2 knockdown to up to 85% (Figure 5D). Importantly, we found that PACS-2 downregulation significantly decreased the extracellular tc-VLP infectivity with up to 10-fold reduction of the titres as compared to tc-VLPs produced in control cells (Figure 5E). Furthermore, we observed more than 10-fold reduction of intracellular infectivity of tc-VLPs produced in PACS-2 down-regulated cells (Figure 5E), which indicated that PACS-2 is important for production of infectious particles at the level of virion assembly.

We sought to confirm this result with full length, live CCHFV (Figure 5F) produced in PACS-2 knockdown *vs*. control cells (Figure 5D). Following infection with virus inoculate, samples from the infected cells and supernatants were collected at 24-hour following infection and analysed by qPCR on viral RNAs. Notably, we found that the extracellular CCHFV RNAs harvested from PACS-2 down-regulated cells displayed *ca*. 10-fold decrease of CCHFV RNAs as compared to controls cells (Figure 5F), which agreed with reduction of intracellular viral RNAs in producer cells (Figure 5F) and confirmed that PACS-2 is a crucial factor for production of infectious CCHFV particles.

Finally, to exclude that PACS-2 could act at the step of virus entry, notably by acting on trafficking of host entry factors, we infected PACS-2 downregulated Huh7.5 cells. We found that tc-VLPs displayed similar levels of infectivity whether PACS-2 was down-regulated or not in target cells (Figure 5G), which agreed with its involvement at the step of assembly of viral particles.

**Figure 6.**
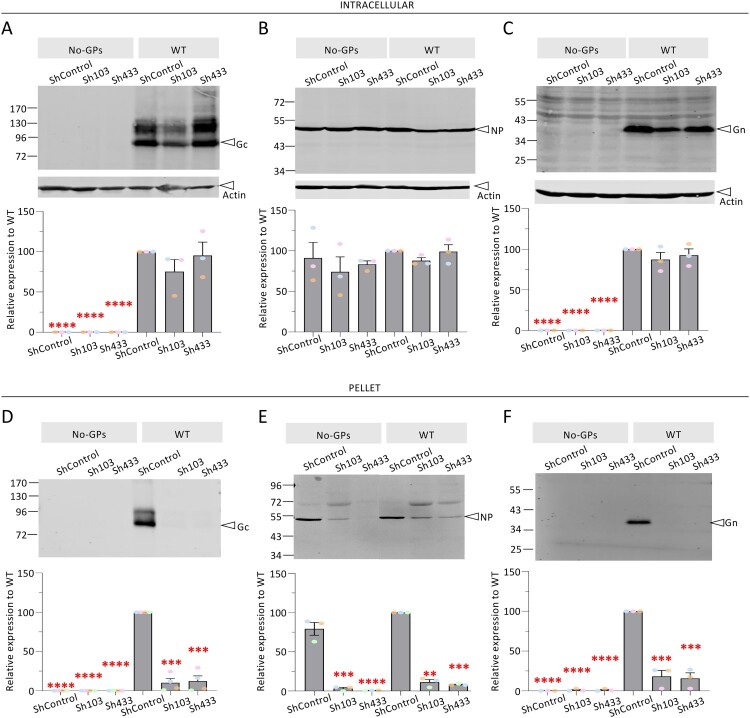
**Viral incorporation of CCHFV GPs in PACS-2 knockdown cells.** Downregulation of PACS-2 was achieved in Huh7.5 cells *via* a lentiviral vector expressing PACS-2 shRNAs (Sh103, Sh433) or control shRNA (ShControl). The cells were then used to produce CCHFV tc-VLPs harbouring WT GPC or no GPC (No-GPs). (**A-C**) Intracellular levels and processing of CCHFV GPs produced in the presence control shRNA *vs*. PACS-2 ShRNA. Representative Western blot analysis and relative quantification of intracellular Gc, NP and Gn compared to WT proteins (lower panels). Protein band intensities were quantified and normalized relative to actin and expressed as fold change compared to WT (lower panels). (**D-F**) Cell supernatants containing tc-VLPs were pelleted by ultracentrifugation through 20% sucrose cushions, resuspended in Opti-MEM medium and analysed by Western blot. Representative Western blot analysis and relative quantification of Gc, NP and Gn expressed as fold change compared to WT proteins (lower panels). Molecular weight markers are marked to the left (kDa). Statistical significance was determined using parametric student-t test compared with WT GPs. Average number of repeats for intracellular CCHFV proteins: Gn = 3, Gc = 3, NP = 3. Average number of repeats for CCHFV proteins in pellets: Gn = 3, Gc = 3, NP = 3. The values are displayed as means ± SEM. Each dot in the graphs corresponds to the value of an individual experiment and dots from one colour are from the same experiment. Statistical significance was determined using parametric student-t test compared with WT protein in ShControl condition. The values are displayed as means ± SEM. Each dot in the graphs corresponds to the value of an individual cell.

When we investigated intracellular expression of CCHFV proteins, no significant effect for NP, Gn and Gc expression could be detected upon PACS-2 down-regulation, as compared to cells expressing a control short hairpin RNA (Figure 6A–C). Yet, when we analysed the pellets of ultracentrifuged supernatants of tc-VLP producer cells, we found that PACS-2 knockdown drastically reduced Gc (by *ca*. 90%) and Gn (by over 85%) secretion (Figure 6D, F). Furthermore, we found that PACS-2 downregulation also resulted in a notable reduction (by over 90%) of the secretion of NP (Figure 6E). These results suggested that PACS-2 modulates trafficking of both CCHFV GPs and NP.

**Figure 7.**
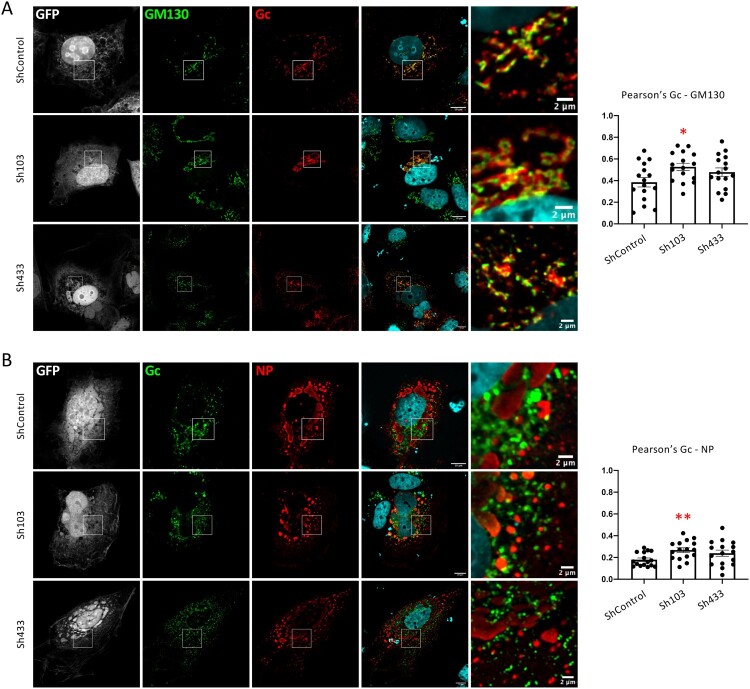
**CCHFV GPs trafficking in PACS-2 knockdown cells.** Downregulation of PACS-2 was achieved in Huh7.5 cells *via* a lentiviral vector expressing PACS-2 shRNAs (Sh103, Sh433) or control shRNA (ShControl). The cells were then used to produce CCHFV tc-VLPs. (**A**) Golgi localization of CCHFV Gc glycoprotein. Confocal microscopy analysis of Huh7.5 cells producing tc-VLPs encoding a GFP marker that were generated with WT GPC. At 48 h post-transfection, cells were fixed, permeabilized with Triton X-100, and stained for GFP (grey channel), Golgi (anti-GM130, green channel), Gc (11E7, red channel), and nuclei (Hoechst, blue channel). (**B**) Gc colocalization with NP. Confocal microscopy analysis of Huh7.5 cells producing tc-VLPs encoding a GFP marker that were generated with WT GPC. At 48 h post-transfection, cells were fixed, permeabilized with Triton X-100, and stained for GFP (grey channel), Gc (11E7, green channel), NP (2B11, red channel), and nuclei (Hoechst, blue channel). Scale bars represent 10 μm. Magnification of the squared areas is shown at the right side of each condition, scale bars from squared area represent 2μm. Pearson’s coefficients were calculated using FIJI (JACoP) and expressed as means ± SEM. Statistical significance was determined using non-parametric two-tailed Mann-Whitney test. The values are displayed as means ± SEM. Each dot in the graphs corresponds to the value of an individual cell.

To confirm these results, we subsequently investigated Gc localization in PACS-2 down-regulated cells. We found that the knockdown of PACS-2 expression induced a slight but significant increased colocalization of Gc with the Golgi marker GM130 (Figure 7A) but did not have any impact on colocalization of Gc with early or late endosomes (Supplementary Fig. 3). These results suggested that the loss of PACS-2 expression does not impact Gc trafficking from PM to Golgi, but impairs Gc trafficking through the Golgi. Interestingly, this result phenocopies the increased colocalization of Gc with GM130 that we observed for Gn AC mutant (Figure 4A, D), albeit to a lesser extent. Furthermore, we found that Gc and NP colocalization was significantly increased in PACS-2 down-regulated cells (Figure 7B), which reflected the accumulation of virion structural components when assembly was blocked.

Finally, the overexpression of PACS-2 in tc-VLP producer cells (Supplementary Fig. 4A) did not raise the production of infectious intracellular and extracellular viral particles (Supplementary Fig. 4B), which agreed with the lack of increased secretion of viral GP components (Supplementary Fig. 4C-H) and suggested that PACS-2 is not a rate limiting assembly factor for envelopment and secretion of CCFHV particles. Note that, in contrast to Gn and Gc GPs, NP secretion was slightly increased by *ca*. 50% (Supplementary Fig. 4G).

Altogether, the above observations underscored that PACS-2 is a critical host factor modulating CCHFV GPs intracellular trafficking and CCHFV assembly and production of infectious particles.

## Discussion

Due to its high pathogenicity, CCHFV requires handling in Biosafety level 4 laboratories (BSL-4), a scarce resource in the world, which makes the study of assembly, envelopment, and secretion of CCHFV particles challenging. By comparison with other Bunyaviruses, it is currently understood that CCHFV assembly occurs at or near Golgi membranes [13]; yet, how viral proteins reach the assembly sites and which host cellular factors are involved remain open questions. One specific feature of the Gc GP of Nairoviruses is its unusually long cytoplasmic tail compared to other Bunyaviruses, which potentially contains different domains that could control distinct trafficking pathways. Taking advantage of the tc-VLP assay [30,31,36], we deleted or mutated several putative trafficking motifs in Gc and Gn cytoplasmic domains to get better insight into CCHFV envelope assembly and secretion of viral particles.

**Figure 8.**
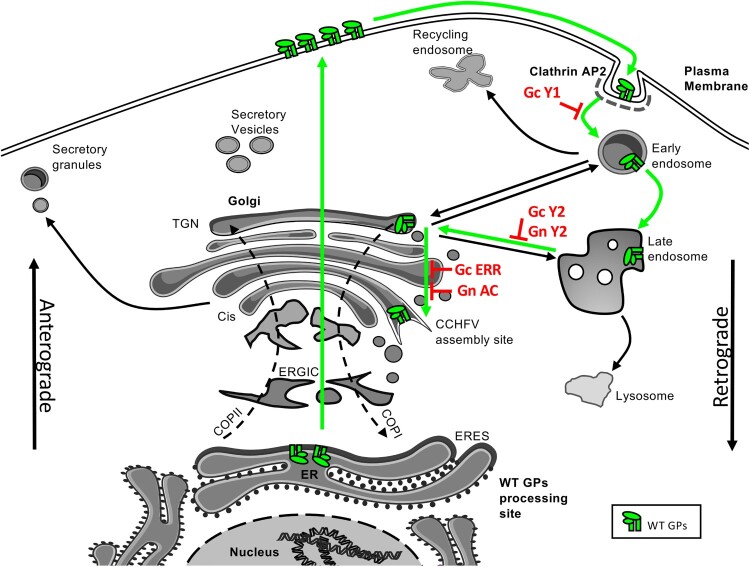
**Working model of CCHFV GP retrograde intracellular trafficking**. The molecular characterization of Gn and Gc CT motifs suggest that after translation, either GP follows the secretion pathway, *via* an unidentified mechanism, to reach the plasma membrane (PM). From the PM, Gc is re-endocytosed *via* its first tyrosine domain (Y1), which probably involves a clathrin-mediated endocytosis *via* AP-2 complexes. After endocytosis, Gn and Gc traffic *via* the endosomal retrograde pathway and are transferred from the late endosomes to the Golgi through the usage of their second tyrosine motif (Gn Y2 and Gc Y2 respectively). From there, Gn and Gc progress along the Golgi stacks to reach the unidentified assembly site, probably *via* COPI binding to Gc ERR motif and Gn AC motif *via* the PACS-2 protein adaptor. Interestingly, our results suggest that NP trafficking and/or assembly into particles is also dependent on PACS-2 protein.

Interestingly, the removal of most (*i.e.* 85%) of the Gc CT (Gc-ΔCT GPC) increased the exposition of the GPs at the plasma membrane, as deduced by the increased formation of syncytia and GP staining in non-permeabilized cells (Supplementary Fig. 2). Moreover, this mutant GP was unable to traffic to virion assembly site and exhibited impaired Gn and Gc (but not NP) secretion as well as infectivity of tc-VLPs. Overall, these results confirmed that the cytoplasmic domains of CCHFV GPs contain important trafficking signals. Accordingly, a more subtle mutant, Gc Y1, that disrupts a tyrosine-based trafficking motif, also exhibited an over-expression of Gc at the plasma membrane (Supplementary Fig. 2), which suggested that traffic to and/or exposure to the plasma membrane is an important step for CCHFV synthesis and which implied that its GPs must return to the Golgi to reach the CCHFV assembly site (Figure 8). Although such back tracking seems counter-intuitive, this anterograde followed by retrograde trafficking is not unique as it is notably used by Golgi resident proteins such as Furin or TGN38 [33]. One possibility is that retrograde trafficking could be essential for both proper maturation of CCHFV GPs and/or encountering/recruitment of viral RNA. Indeed, as the final maturation step of Gc involves the cleavage of preGc by an uncharacterized SKI-1/S1P protease [10], one cannot exclude that this cleavage event takes place in endosomal/lysosomal compartments since the involved proprotein convertases are active in a wide range of compartments [41]. On the other hand, cytoplasmic “condensates” that contain viral RNA and NP have been observed in CCHFV-infected cells and may represent replication sites [42], raising the possibility that Gn/Gc complex needs to use specific intracellular pathways to interact with and transport the viral RNA through Gn zinc finger domains to the assembly sites.

The above observations concerning CCHFV GPs led us first to investigate the functions of all the trafficking motifs that were detected in both Gn and Gc cytosolic domains.

Particularly, the last 5 amino acids of Gc (KTHIG), whose deletion abrogated production of infectious CCHFV tc-VLPs, would fit with a non-canonical di-lysine motif KxHxx as an ER retrieval (ERR) motif. Such a motif was first identified within an alternative viral envelope GP, the spike protein of SARS-CoV-1 [35]. Curiously, while dibasic motifs are absent in Gc proteins from Orthobunyaviruses and Tospoviruses [20], they can be detected in Gc from Phleboviruses (KKxx) and Hantaviruses (KKxx or KxKxx), suggesting different GP trafficking requirements among Bunyaviruses. These dibasic motifs (KKxx or KxKxx), present in some type I transmembrane proteins, are recognized by the COPI coatomer to achieve their retrograde transport from TGN to ER [28]. The presence of such a motif in Gc CT therefore suggests that Gc needs this motif to reach a dedicated place, probably the assembly site (Figure 8). This may be analogous to the spike protein of coronaviruses which needs to return to the ER-Golgi Intermediate Compartment (ERGIC) to allow its incorporation into virions [43].

We also identified four putative tyrosine-based motifs, two located on Gn (YREL and YLNL, Gn Y1 and Y2 motifs, respectively) and two located on Gc (YRHL and YRRI, Gc Y1 and Y2 motifs, respectively). Tyrosine-based motifs YXXΦ are known to be involved in protein recycling from the plasma membrane *via* clathrin-mediated endocytosis [44] and in protein targeting to the lysosomes [45], endosomal compartments [46] and TGN [47]. YXXΦ motifs bind to the µ subunit of adaptor protein complexes (APs) with the highest avidity for µ2 of AP-2 complex [33].

Interestingly, our results identified distinct functional features for these different tyrosine-based motifs. As above discussed, mutations of Gc Y1 showed increased exposition of Gc at the plasma membrane, hence suggesting that this motif acts as classical endocytosis motif (Figure 8). Yet, Gn Y1 as well as Gc Y1 mutations resulted in only a mild defect, of up to two-fold of viral incorporation of either GP and had no impact on tc-VLP infectivity or on Gc localization at Golgi. This suggested that the bulk of envelope GPs were still able to reach assembly sites, owing to alternative trafficking signals or to a still active, though weaker, endocytosis mechanism, and that either motif could be important for removing exceeding GP from the plasma membrane rather than to be a limiting factor for CCHFV envelopment. In contrast to the above, the two other tyrosine-based motifs located on either envelope GP, *i.e.* Gn Y2 and Gc Y2, were shown to be crucial for virion assembly. Indeed, their individual mutation strongly impaired Gn and Gc secretion, virion envelopment, and tc-VLP infectivity. Furthermore, this correlated with prevention of Gc colocalization at the Golgi, and consequently, increased Gc colocalization with late endosomes. These results suggested that these mutant GPs have impaired traffic between endosomes and Golgi (Figure 8), and thus, that Gn and Gc Y2 tyrosine-based motifs represent crucial GP intracellular trafficking domains. Notably, it was previously suggested that tyrosine-based motifs could also act as late domains to recruit host cellular factors necessary to complete virion budding and to compensate the lack of matrix in Bunyaviruses [20]; yet, one would expect that these mutants could be blocked within the assembly sites (Golgi) and would have altered NP secretion, which was not observed in our assays.

Additionally, we identified two functional dileucine motifs either in the cytosolic domain of Gn or in the cytoplasmic tail of Gc. Di-leucine based motifs are another class of sorting motifs that act in the TGN/endosomal system. They have been shown to be recognized by AP-1, AP-2 and AP-3 [26] to mediate different sorting events, such as internalization from plasma membrane and sorting to endosomes [27]. Curiously, the substitution for alanines of a di-leucine motif in Gc strongly impaired Uukuniemi virus (UUKV) VLPs budding, suggesting that this motif would act between assembly and secretion of UUKV VLPs [21]. In our study, we discovered that the disruption of the dileucine motif in the cytoplasmic domains of both the Gn or Gc proteins resulted in a deficiency in the envelopment and secretion of CCHFV tc-VLPs. Furthermore, our confocal microscopy analysis only revealed a slight impairment of the colocalization of Gc of the Gn LL mutant with the early endosome marker (Rab5), which is likely not significant for CCHFV assembly owing to very low Pearson’s coefficients for WT GPs. Thus, these results suggested that the di-leucine motifs identified in CCHFV GPs either may not be trafficking motifs *per se,* or at least may not be involved in the endosomal retrograde pathway. Indeed, consensus di-leucine motifs exhibit a negatively charged amino-acid in position – 2 or – 3 (DXXLL and [DE]XXXL[LI] respectively [33]) which is absent in CCHFV GPs. Yet, as the mutation of these LL motifs dramatically impaired tc-VLP assembly, further work would be necessary to identify at which step do they act, such as *e.g.* Gn/Gc heterodimerization, envelopment or genome packaging.

Finally, we identified putative acidic cluster (AC) motifs, which are composed of a stretch of negatively charged amino acids, in both Gn and Gc cytosolic domains [33]. While mutation of the acidic cluster of Gc GP did not seemingly alter its functions, the exchange of charged amino acids by neutral structural homologs in the acidic cluster of Gn GP (*i.e.* substitution of EKVEETEL for QKVQQTQ) resulted in a complete loss of Gn/Gc secretion as well as of tc-VLP production and infectivity (Figure 8).

Acidic cluster motifs are often found in transmembrane proprotein convertases, such as PC6B and PC7 that are localized to the TGN and were first described in the cytosolic tail of Furin [34]. Interestingly, several viral proteins also contain such motifs including GP of KSHV [48], GP B of HCMV [49], Nef of HIV-1 [50] or TM GP of RD114 virus [39]. These acidic cluster motifs are recognized by PACS (phosphofurin acidic cluster sorting) proteins to achieve intracellular trafficking. The PACS family comprises two members, PACS-1 and PACS-2, that have similar structures but different functions [29]. The structure of PACS proteins consist of a cargo (furin)-binding region (FBR), a disordered middle region (MR) and a C-terminal region (CTR) [29]. Both proteins share 54% sequence identity, but the FBR domain of PACS-2 has 81% sequence identity with that of PACS-1 and is believed to be critical for identifying and binding cargo proteins [29]. Widely expressed in human tissues, the biological function of PACS proteins is to sort client proteins from different organelles. The PACS-1 FBR interacts with the AP-1 and AP-3 adaptor complexes [51] and has been shown to direct the retrograde transport of numerous cellular proteins from endosomal pathway to TGN, including Furin and the cation-independent mannose-6-phosphate receptor (CI-MPR) [52], or viral proteins such as GP B of HCMV [49], the TM protein of RD114 [39], or the accessory protein Nef of HIV-1 [50]. The PACS-2 FBR binds to COPI and controls the ER localization of polycystin-2 [53] and calnexin [54], and the cell surface exposition of the metalloproteinase ADAM17 [55], but also interacts with HIV-1 Nef to trigger MHC-1 down-regulation [40]. Importantly, acidic clusters often contain serine or threonine residues that can be phosphorylated by CK2 (casein kinase 2), which confer different subcellular distribution of the cargo proteins depending on their phosphorylated state.

Our results with mutations of AC motif in CCHFV Gn GP led us to hypothesize that PACS-1 or PACS-2 could be involved in CCHFV life cycle through recognition of the Gn AC motif. Yet, we did not observe any effect neither on tc-VLP nor on live CCHFV infectivity upon PACS-1 silencing, suggesting that PACS-1 is not a crucial cellular host factor involved in production of infectious CCHFV particles.

Importantly, PACS-2 down-regulation severely impaired Gn/Gc secretion and infectivity of tc-VLPs as well as CCHFV genome expression by virus-infected cells, confirming that PACS-2 is an important proviral factor acting at the assembly/secretion level. Since silencing of PACS-2 also impaired the formation of intracellular infectious tc-VLPs, these data suggested that PACS-2 acts at assembly steps rather than secretion of CCHFV particle. Interestingly, we also observed a defect in NP secretion upon PACS-2 down-regulation. Little is known about the ligands of PACS-2, but due to its high homology with PACS-1 [29], one can imagine that PACS-2 could link several proteins at the same time, alike PACS-1 that was shown to bind within same complexes CI-MPR, CK2 and GGA3 *via* different domains contained in the FBR region [56]. It is thus tempting to speculate that PACS-2 could bind Gn *via* its acidic cluster and NP *via* an unidentified domain. This tripartite association could then help the encountering of NP with Gn or with the Gn/Gc complex, or, alternatively, could allow the co-trafficking of CCHFV GPs and NP to the assembly site. The latter would explain why the mutation of the Gn AC affects only the secretion of Gn/Gc (Figure 8), while the absence of PACS-2 impairs the secretion of both Gn/Gc and NP. Finally, we found that the mutation of the AC motif in CCHFV Gn GP or the depletion of PACS-2 impaired the trafficking of Gc GP through the Golgi and the colocalization of Gc and NP. Since PACS-2 has been shown to adapt AC-containing proteins to COPI [53], it is reasonable to hypothesize that PACS-2 connects Gn-Gc-NP complex to COPI along the retrograde pathway through the Golgi to ultimately reach the assembly site.

In conclusion, we show that various viral determinants on CCHFV GPs and the host factor PACS-2 can induce a stepwise CCHFV GPs assembly/incorporation, egress and secretion of infectious particles.

## Materials and methods

**Cell culture and reagents.** Huh-7.5 hepato-carcinoma (kind gift from C. Rice) and HEK 293 T kidney cells were grown in Dulbecco’s modified Eagle’s medium (DMEM) complemented with 10% fetal bovine serum (FBS) and 1% penicillin–streptomycin. All the cells were grown in a 37°C and 5% CO_2_ incubator.

**Plasmids and constructs.** The constructs encoding wild-type CCHFV strain IbAr10200 L polymerase (pCAGGS-V5-L), CCHFV nucleoprotein NP (pCAGGS-NP), CCHFV specific eGFP-expressing minigenome (pT7RiboSM2_vL_eGFP), T7 RNA polymerase (pCAGGS-T7), CCHFV M-segment polyprotein (pCAGGS-GPC), and an empty vector without viral genes (pCAGGS) were described previously [30,36,57]. Several CCHFV M segment cDNA mutants were generated using standard molecular cloning techniques and confirmed by DNA sequencing. Standard PCR and oligonucleotide-specific mutagenesis reactions were carried out with Phusion enzyme (NEBio-labs). Cytoplasmic tail mutations in Gc or Gn were introduced in the context of expression of the whole GPC. By site-directed mutagenesis, Gc tyrosine motif mutant one Y1 (YRHL/ARHL) was created by changing the tyrosine to alanine. Gc tyrosine motif mutant two Y2 (YRRI/ARRI) was generated by changing tyrosine to alanine. Gc dileucine motif (LL/AA) mutant was generated by mutagenesis. Acidic motif mutations were created (DDEE/NNQQ). ERR mutation in Gc cytoplasmic tail of GPC was generated by deletion of its last residues (ΔKTHIG). The removal of the Gc cytoplasmic tail mutant was generated as described previously [58] for the Gc ΔCT-expressing GPC plasmid (ΔCT). Similarly, by using site-directed mutagenesis, Gn tyrosine motif mutant one Y1 (YREL/AREL) was created by changing the tyrosine to alanine. Gn tyrosine motif mutant two Y2 (YLNL/ALNL) was generated by changing tyrosine to alanine. Gn dileucine motif (LL/AA) mutant was generated by mutagenesis. Acidic motif mutations in Gn were created (EKVEETE/QKVQQTQ). Details of oligonucleotides used for the constructs are available upon request. The pCAG-PACS-2-HA construct was derived from pcDNA-PACS-2-HA (kind gift of G. Thomas) [53]. Briefly, the hCMV promoter of pcDNA-PACS-2-HA was exchanged by the CAG promoter of pCAGGS-NP construct via SnaBI/EcoRI restriction and ligation following classical cloning strategy.

**Antibodies.** Anti-PreGc (clone 11E7) and anti-NP (clones 9D5 for Western blot analysis and 2B11 for immunofluorescence analysis) mouse monoclonal antibodies targeting CCHFV were received from the Joel M. Dalrymple-Clarence J. Peters USAMRIID Antibody Collection through BEI Resources, NIAID, NIH. Anti-Gn rabbit polyclonal antibody was kindly given by Ali Mirazimi (Karolinska Institute, Sweden). Monoclonal mouse anti-β-actin AC-74 (Sigma), anti-HA (3F10, Sigma), anti-PACS-1 (A12659, ABclonal), anti-PACS-2 (ab222316, Abcam) were used at 1/1,000 for Western blotting. Anti-GM130 (ab52649, Abcam), anti-Rab5 (C8B1, Cell Signaling) and anti-Rab7 (D95F2, Cell Signaling) were used at 1/200 for immunofluorescence. All the antibodies were used according to the manufacturer’s guidelines.

**Production and titration of full-length CCHFV particles.** To generate a titrated stock of live CCHFV, Huh-7.5 cells were infected in the BSL-4 laboratory (Jean Mérieux, Lyon) using CCHFV isolate IbAr10200 (obtained from Institut Pasteur) at a multiplicity of infection (MOI) of 0.01 and the production was harvested 24 h post-infection. Infectious titre was determined by NP immunostaining on VeroE6 cells.

At 24 h post-infection of Huh-7.5 cells WT or KD for PACS-1 or PACS-2 (see below) at MOI = 0.01 with this reference stock, cells and supernatants were harvested and lysed with TriReagent (Molecular Research Center, Inc. Cat. No. TR118). RNAs were extracted according to the manufacturer’s protocol and level of viral RNA was determined by RT-qPCR (see below).

**Knockdown of PACS-1 and PACS-2.** Expression of specific shRNAs AGATCTGTTCAGTCGCTC through a lentiviral vector induced the PACS-1 downregulation in producer cells [39]. Two PACS-2 shRNAs were ordered from SIGMA-ALDRICH, ref sequence NM_015197, TRCN000135103 (sh103) (target sequence: CACCAAGGAGAAGAACAAGAA) and TRCN0000137443 (sh433) (target sequence: GACCAGGCAACAGAACTTCAA). To package shRNA-expressing lentiviral vectors, HEK293 T cells were seeded in 10-cm plates and were transfected with 8 µg of the FG12 GFP/shRNA construct or pLKO PACS-2 construct, 8 µg of the HIV packaging construct psPAX2, and 2.7 µg of the VSV-G glycoprotein construct phCMV-G. The vector particles were collected 48 h post-transfection by 0.45 µm filters and aliquots were kept at – 80°C for further use. The infectious titres were determined on 293 T cells by FACS or VCN analysis. To downregulate PACS-1 and PACS-2 in Huh7.5 cells, the shRNA lentiviral vectors were used at a MOI of 10. For PACS-1 knockdown, two days after transduction, the transduced Huh7.5 cells were seeded in 10-cm plates and transfected 24 h post-seeding with plasmids allowing production of WT tc-VLPs to perform biochemical assay either in control or knockdown cells. Similarly, for PACS-2 knockdown, 7 days after transduction, cells were used for further transfections to generate WT tc-VLPs in knockdown or control cells to perform biochemical assays. In parallel, a lentiviral vector without shRNA was used as a control. The knockdown of endogenous PACS-1 and PACS-2 was validated by immunoblotting.

For full-length virus, Huh-7.5 cells were infected after 2 days of transduction for PACS-1 or 7 days of transduction of PACS-2.

**Production of tc-VLPs.** Huh7.5 cells were seeded in 10 cm dishes and transfected with 3.6 µg of pCAGGS-V5-L, 1.2 µg of pCAGGS-NP, 1.2 µg of pT7riboSM2-vS-GFP, 3 µg of pCAGGS-GP, 3 µg of pCAGGS-T7 by using GeneJammer transfection reagent (Agilent), as previously described [30,36]. pCAGGS plasmid was additionally transfected to keep the DNA amount uniform. For PACS-2 over-expression experiments, increasing amount of pCAG-PACS-2-HA construct, complemented or not with pCAGGS plasmid to achieve a total of 8 µg DNA, were added to the above DNA mix prior to the transfection. The transfection media was replaced after 6 h post-transfection. Cells supernatants were harvested 72 h post-transfection and filtered through a 0.45 μm filters and concentrated through ultracentrifugation through a 20% sucrose cushion (SW41 rotor at 28,000 rpm, 2 h, 4°C).

**Intracellular and extracellular infectivity of tc-VLPs.** Infectivity was analysed infection of pre-transfected Huh7.5 cells expressing L and NP plasmid on Huh7.5 cells. For titration, Huh-7.5 cells were pre-transfected with 2.4 µg of pCAGGS-V5-L and 4.8 µg of pCAGGS-NP using GeneJammer transfection reagent. The transfection medium was replaced at 6 h post-transfection and cells were seeded in 24-well plates in DMEM. Twenty-four hours post pre-transfection, cells were infected, as previously described [30,36]. Intracellular tc-VLP particles were released upon producer cells lysis by three repeated freeze–thaw cycles, followed by clarification by centrifugation. At 24 h post-infection, the infectivity was analysed by flow cytometry (FACS) using a MACSQuant (Miltenyi Biotec) VYB apparatus. Data were then analysed using the FlowJo software. Titres were calculated according to the formula (number of seeded Huh7.5 cells × % of GFP-positive cells) × 1000/μl of inoculum and expressed as infectious units per ml of supernatant (extracellular infectivity) or cell lysate (intracellular infectivity).

**RNA extraction and RT-qPCR analysis**. Viral RNAs were extracted either from infected cell (intracellular) or from the supernatant (extracellular) using TRI Reagent according to the manu-facturer’s instructions (Molecular Research Center). RNAs were reverse transcribed using random oligonucleotide primers with iScript (Bio-Rad). The following specific primers against S segment were used: for CCHFV RNA quantification, forward primer 5’ – TGTTGCCTCCACCAGAGCA and reverse primer 5’-TTCCAAATGGCCAGTGCC [59]. Quantitative PCR (qPCR) was performed using FastStart Universal SYBR Green Master (Roche) on a StepOne Real-Time PCR System (Applied Biosystems). As an internal control of extraction, in vitro-transcribed exogenous RNAs from the linearized Triplescript plasmid pTRI-Xef (Invitrogen) were added to the samples prior to RNA extraction and quantified with specific primers (5’-CGACGTTGTCACCGGGCACG and 5’-ACCAGGCATGGTGGTTACCTTTGC). All values of intracellular CCHFV RNAs were normalized to glyceraldehyde 3-phosphate dehydrogenase (GAPDH) gene transcription. For GAPDH mRNA quantification, we used the forward 5’-AGGTGAAGGTCGGAGTCAACG and reverse 5’-TGGAAGATGGTGATGGGATTTC primers. All extracellular CCHFV RNAs were normalized to XEF signals.

**Western blot analysis.** Huh7.5 transfected cells were washed with cold PBS and detached with Versene (Gibco). Cells were lysed in cell lysate buffer (20 mM Tris pH 7.5, 1% Triton, SDS 0.05%, sodium Deoxycholate Acid 0.5% and 150 mM NaCl) containing protease inhibitors (Roche). Cell lysates were clarified by centrifugation at 13,000 rpm for 30 min at 4°C. Pellets from ultracentrifuged supernatants were resuspended in Gibco Opti-MEM medium in 1/100 of the initial volumes. To detect the expression of NP, GC, Gn, PACS-1, PACS-2 and Actin, samples were denatured at 95°C for 5 min in reducing loading buffer (5X Blue Loading Buffer, 200 mM Tris HCl pH6.8, 10% SDS, 500 mM β-mercaptoethanol and 50% glycerol) and electrophoresed on 10% polyacrylamide gels. Alternatively, for detection of Gc, samples were processed in a non-reducing loading buffer (5X Blue Loading Buffer, 200 mM Tris HCl pH6.8, 5% SDS, and 50% glycerol) and electrophoresed on 10% polyacrylamide gels. Proteins were transferred to a nitrocellulose membrane by electro blotting (Bio-Rad) for 1 h at 100 V. The membrane was incubated in TBST (20 mM Tris HCl, pH 7.5, 150 mM NaCl and Tween 0.05%)-milk 5% for 1 h. Nitrocellulose membranes were incubated overnight at 4°C with primary antibodies for the detection of CCHFV protein (mouse monoclonal 9D5 anti-NP (1:1,000), mouse monoclonal 11E7 anti-Gc (1:500) and rabbit polyclonal anti-Gn (1:5,000)) diluted in TBST-milk 5%. After 3 washes using TBST, then membranes were incubated with immunofluorophore-labelled secondary antibodies (LiCor Biosciences) at 1:10000 dilution in TBST for 1 h at room temperature, followed by imaging with Odyssey infrared imaging CLx system (LiCor Biosciences). Quantification of proteins was performed with Odyssey imaging CLx system software.

**Immunofluorescence analysis.** The procedure was described previously [60]. Briefly, Huh7.5 cells were seeded in 6-well plates on coverslips and transfected with the plasmids described above. The transfection media was replaced after 6 h post-transfection and infected with WT tc-VLPs at 24 h post-transfection. Forty-eight hours post-transfection, the cells were fixed with 4% paraformaldehyde (PFA) for 15 min at room temperature. Next, unless otherwise specified, the cells were permeabilized with 0.1% Triton X-100 for 7 min. Cells were washed 3 times with PBS and incubated for 1 h at room temperature with primary antibodies diluted in PBS/1% BSA. After 3 washes with PBS/1% BSA, cells were further incubated with Alexa Fluor-conjugated secondary antibodies (Alexa Fluor 568 and Alexa Fluor 647; Thermo Fisher) in PBS/1% BSA. After 3 washes with PBS, nuclei were stained with Hoechst 33342 (Molecular Probes), and the coverslips were mounted with Mowiol 40–88 (Sigma-Aldrich). The slides were examined using a confocal microscope LSM-800 (Zeiss). Pearson’s correlation coefficients were calculated using FIJI (JACoP) and were calculated on n cells from 3 separate experiments and expressed as mean ± SEM.

**Statistical analysis.** All the statistical analysis were performed using GraphPad Prism version 5.02 for Windows or Mac, GraphPad Software (San Diego, California, USA). For statistical comparisons, the Mann–Whitney or the student-t test were used. For statistical analysis, a *p*-value of 0.05 or less was considered significant. Data are presented as mean ± standard error of the mean (SEM), and results of the statistical analysis are shown as follows: ns, not significant (*P* > 0.05); *, *P* < 0.05; **, *P* < 0.01; and ***, *P* < 0.001.

## Supplementary Material

Supplementary_Material_Hot_winters_precede_more_intense_WNV_clean

## References

[1] Hoogstraal H. The epidemiology of tick-borne Crimean-Congo hemorrhagic fever in Asia, Europe, and Africa. J Med Entomol. 1979 May 22;15(4):307–417. doi:10.1093/jmedent/15.4.307 113533

[2] Simpson DI, Knight EM, Courtois G, et al. Congo virus: a hitherto undescribed virus occurring in Africa. I. Human isolations–clinical notes. East Afr Med J. 1967 Feb;44(2):86–92. 6040759

[3] Messina JP, Pigott DM, Golding N, et al. The global distribution of Crimean-Congo hemorrhagic fever. Trans R Soc Trop Med Hyg. 2015 Aug;109(8):503–513. doi:10.1093/trstmh/trv050 26142451 PMC4501401

[4] Freitas N, Legros V, Cosset FL. Crimean-Congo hemorrhagic fever: a growing threat to Europe. C R Biol. 2022 May 11;345(1):17–36. doi:10.5802/crbiol.78 35787618

[5] Bernard C, Joly Kukla C, Rakotoarivony I, et al. Detection of Crimean-Congo haemorrhagic fever virus in Hyalomma marginatum ticks, southern France, May 2022 and April 2023. Euro Surveill. 2024 Feb;29(6):2400023. doi:10.2807/1560-7917.ES.2024.29.6.2400023 38333936 PMC10853980

[6] Gargili A, Estrada-Pena A, Spengler JR, et al. The role of ticks in the maintenance and transmission of Crimean-Congo hemorrhagic fever virus: A review of published field and laboratory studies. Antiviral Res. 2017 Aug;144:93–119. doi:10.1016/j.antiviral.2017.05.010 28579441 PMC6047067

[7] Spengler JR, Bergeron E, Rollin PE. Seroepidemiological Studies of Crimean-Congo Hemorrhagic Fever Virus in Domestic and Wild Animals. PLoS Negl Trop Dis. 2016 Jan;10(1):e0004210. doi:10.1371/journal.pntd.0004210 26741652 PMC4704823

[8] Altamura LA, Bertolotti-Ciarlet A, Teigler J, et al. Identification of a novel C-terminal cleavage of Crimean-Congo hemorrhagic fever virus PreGN that leads to generation of an NSM protein. J Virol. 2007 Jun;81(12):6632–6642. doi:10.1128/JVI.02730-06 17409136 PMC1900101

[9] Sanchez AJ, Vincent MJ, Nichol ST. Characterization of the glycoproteins of Crimean-Congo hemorrhagic fever virus. J Virol. 2002 Jul;76(14):7263–7275. doi:10.1128/JVI.76.14.7263-7275.2002 12072526 PMC136317

[10] Vincent MJ, Sanchez AJ, Erickson BR, et al. Crimean-Congo Hemorrhagic Fever Virus Glycoprotein Proteolytic Processing by Subtilase SKI-1. J Virol. 2003 Aug;77(16):8640–8649. 12885882 10.1128/JVI.77.16.8640-8649.2003 PMC167219

[11] Du S, Peng R, Xu W, et al. Cryo-EM structure of severe fever with thrombocytopenia syndrome virus. Nat Commun. 2023 Oct 10;14(1):6333. doi:10.1038/s41467-023-41804-7 37816705 PMC10564799

[12] Hover S, Charlton FW, Hellert J, et al. Organisation of the orthobunyavirus tripodal spike and the structural changes induced by low pH and K(+) during entry. Nat Commun. 2023 Sep 21;14(1):5885. doi:10.1038/s41467-023-41205-w 37735161 PMC10514341

[13] Spiegel M, Plegge T, Pohlmann S. The Role of Phlebovirus Glycoproteins in Viral Entry, Assembly and Release. Viruses. 2016 Jul 21;8(7):202. doi:10.3390/v8070202 27455305 PMC4974537

[14] Cole NB, Lippincott-Schwartz J. Organization of organelles and membrane traffic by microtubules. Curr Opin Cell Biol. 1995 Feb;7(1):55–64. doi:10.1016/0955-0674(95)80045-X 7755990

[15] Schafer W, Stroh A, Berghofer S, et al. Two independent targeting signals in the cytoplasmic domain determine trans-Golgi network localization and endosomal trafficking of the proprotein convertase furin. EMBO J. 1995 Jun 1;14(11):2424–2435. doi:10.1002/j.1460-2075.1995.tb07240.x 7781597 PMC398356

[16] Schekman R, Orci L. Coat proteins and vesicle budding. Science. 1996 Mar 15;271(5255):1526–1533. doi:10.1126/science.271.5255.1526 8599108

[17] Beitia Ortiz de Zarate I, Kaelin K, Rozenberg F. Effects of mutations in the cytoplasmic domain of herpes simplex virus type 1 glycoprotein B on intracellular transport and infectivity. J Virol. 2004 Feb;78(3):1540–1551. doi:10.1128/JVI.78.3.1540-1551.2004 14722308 PMC321396

[18] Byland R, Vance PJ, Hoxie JA, et al. A conserved dileucine motif mediates clathrin and AP-2-dependent endocytosis of the HIV-1 envelope protein. Mol Biol Cell. 2007 Feb;18(2):414–425. doi:10.1091/mbc.e06-06-0535 17108326 PMC1783771

[19] Lontok E, Corse E, Machamer CE. Intracellular targeting signals contribute to localization of coronavirus spike proteins near the virus assembly site. J Virol. 2004 Jun;78(11):5913–5922. doi:10.1128/JVI.78.11.5913-5922.2004 15140989 PMC415842

[20] Strandin T, Hepojoki J, Vaheri A. Cytoplasmic tails of bunyavirus Gn glycoproteins-Could they act as matrix protein surrogates? Virology. 2013;437:73–80. doi:10.1016/j.virol.2013.01.001 23357734

[21] Overby AK, Popov VL, Pettersson RF, et al. The cytoplasmic tails of Uukuniemi Virus (Bunyaviridae) G(N) and G(C) glycoproteins are important for intracellular targeting and the budding of virus-like particles. J Virol. 2007 Oct;81(20):11381–11391. doi:10.1128/JVI.00767-07 17670814 PMC2045573

[22] Haferkamp S, Fernando L, Schwarz TF, et al. Intracellular localization of Crimean-Congo Hemorrhagic Fever (CCHF) virus glycoproteins. Virol J. 2005 Apr 25;2:42. doi:10.1186/1743-422X-2-42 15850490 PMC1090624

[23] Estrada DF, Boudreaux DM, Zhong D, et al. The Hantavirus Glycoprotein G1 Tail Contains Dual CCHC-type Classical Zinc Fingers. J Biol Chem. 2009 Mar 27;284(13):8654–8660. doi:10.1074/jbc.M808081200 19179334 PMC2659224

[24] Mishra AK, Hellert J, Freitas N, et al. Structural basis of synergistic neutralization of Crimean-Congo hemorrhagic fever virus by human antibodies. Science. 2022 Jan 7;375(6576):104–109. doi:10.1126/science.abl6502 34793197 PMC9771711

[25] Guardado-Calvo P, Rey FA. The Viral Class II Membrane Fusion Machinery: Divergent Evolution from an Ancestral Heterodimer. Viruses. 2021 Nov 26;13(12):2368. doi:10.3390/v13122368 34960636 PMC8706100

[26] Rapoport I, Chen YC, Cupers P, et al. Dileucine-based sorting signals bind to the beta chain of AP-1 at a site distinct and regulated differently from the tyrosine-based motif-binding site. EMBO J. 1998 Apr 15;17(8):2148–2155. doi:10.1093/emboj/17.8.2148 9545228 PMC1170559

[27] Rohn WM, Rouille Y, Waguri S, et al. Bi-directional trafficking between the trans-Golgi network and the endosomal/lysosomal system. J Cell Sci. 2000 Jun;113(Pt 12):2093–2101. doi:10.1242/jcs.113.12.2093 10825282

[28] Ma W, Goldberg J. Rules for the recognition of dilysine retrieval motifs by coatomer. EMBO J. 2013;32:926–937. doi:10.1038/emboj.2013.41 23481256 PMC3616288

[29] Thomas G, Aslan JE, Thomas L, et al. Caught in the act - protein adaptation and the expanding roles of the PACS proteins in tissue homeostasis and disease. J Cell Sci. 2017 Jun 1;130(11):1865–1876. 28476937 10.1242/jcs.199463 PMC5482974

[30] Devignot S, Bergeron E, Nichol S, et al. A virus-like particle system identifies the endonuclease domain of Crimean-Congo hemorrhagic fever virus. J Virol. 2015 Jun;89(11):5957–5967. doi:10.1128/JVI.03691-14 25810550 PMC4442449

[31] Zivcec M, Scholte FE, Spiropoulou CF, et al. Molecular Insights into Crimean-Congo Hemorrhagic Fever Virus. Viruses. 2016 Apr 21;8(4):106. doi:10.3390/v8040106 27110812 PMC4848600

[32] Dell'Angelica EC, Bonifacino JS. Coatopathies: Genetic Disorders of Protein Coats. Annu Rev Cell Dev Biol. 2019 Oct 6;35:131–168. doi:10.1146/annurev-cellbio-100818-125234 31399000 PMC7310445

[33] Bonifacino JS, Traub LM. Signals for sorting of transmembrane proteins to endosomes and lysosomes. Annu Rev Biochem. 2003;72:395–447. doi:10.1146/annurev.biochem.72.121801.161800 12651740

[34] Voorhees P, Deignan E, Van Donselaar E, et al. An acidic sequence within the cytoplasmic domain of furin functions as a determinant of trans-Golgi network localization and internalization from the cell surface. EMBO J. 1995;14:4961–4975. doi:10.1002/j.1460-2075.1995.tb00179.x 7588625 PMC394599

[35] Lontok E, Corse E, Machamer CE. Intracellular Targeting Signals Contribute to Localization of Coronavirus Spike Proteins near the Virus Assembly Site. J Virol. 2004;78:5913–5922. doi:10.1128/JVI.78.11.5913-5922.2004 15140989 PMC415842

[36] Freitas N, Enguehard M, Denolly S, et al. The interplays between Crimean-Congo hemorrhagic fever virus (CCHFV) M segment-encoded accessory proteins and structural proteins promote virus assembly and infectivity. PLoS Pathog. 2020 Sep;16(9):e1008850. doi:10.1371/journal.ppat.1008850 32956404 PMC7529341

[37] Maxfield FR, McGraw TE. Endocytic recycling. Nat Rev Mol Cell Biol. 2004 Feb;5(2):121–132. doi:10.1038/nrm1315 15040445

[38] Bertolotti-Ciarlet A, Smith J, Strecker K, et al. Cellular localization and antigenic characterization of crimean-congo hemorrhagic fever virus glycoproteins. J Virol. 2005 May;79(10):6152–6161. doi:10.1128/JVI.79.10.6152-6161.2005 15858000 PMC1091677

[39] Bouard D, Sandrin V, Boson B, et al. An Acidic Cluster of the Cytoplasmic Tail of the RD114 Virus Glycoprotein Controls Assembly of Retroviral Envelopes. Traffic (Copenhagen, Denmark). 2007;8:835–847. doi:10.1111/j.1600-0854.2007.00581.x 17547695

[40] Atkins KM, Thomas L, Youker RT, et al. HIV-1 Nef binds PACS-2 to assemble a multikinase cascade that triggers major histocompatibility complex class I (MHC-I) down-regulation: analysis using short interfering RNA and knock-out mice. J Biol Chem. 2008 Apr 25;283(17):11772–11784. doi:10.1074/jbc.M707572200 18296443 PMC2431057

[41] Cendron L, Rothenberger S, Cassari L, et al. Proprotein convertases regulate trafficking and maturation of key proteins within the secretory pathway. Adv Protein Chem Struct Biol. 2023;133:1–54. doi:10.1016/bs.apcsb.2022.10.001 36707198

[42] Andersson C, Henriksson S, Magnusson KE, et al. In situ rolling circle amplification detection of Crimean Congo hemorrhagic fever virus (CCHFV) complementary and viral RNA. Virology. 2012;426:87–92. doi:10.1016/j.virol.2012.01.032 22341783

[43] Ujike M, Taguchi F. Incorporation of spike and membrane glycoproteins into coronavirus virions. Viruses. 2015;7:1700–1725. doi:10.3390/v7041700 25855243 PMC4411675

[44] Trowbridge IS, Collawn JF, Hopkins CR. Signal-Dependent Membrane Protein Trafficking in the Endocytic Pathway. Annu Rev Cell Biol. 1993;9:129–161. doi:10.1146/annurev.cb.09.110193.001021 8280459

[45] Marks MS, Roche PA, Van Donselaar E, et al. A lysosomal targeting signal in the cytoplasmic tail of the β chain directs HLA-DM to MHC class II compartments. J Cell Biol. 1995;131:351–369. doi:10.1083/jcb.131.2.351 7593164 PMC2199989

[46] Jackson MR, Nilsson T, Peterson PA. Retrieval of transmembrane proteins to the endoplasmic reticulum. J Cell Biol. 1993;121:317–333. doi:10.1083/jcb.121.2.317 8468349 PMC2200111

[47] Bos K, Wraight C, Stanley KK. TGN38 is maintained in the trans-Golgi network by a tyrosine-containing motif in the cytoplasmic domain. EMBO J. 1993;12:2219–2228. doi:10.1002/j.1460-2075.1993.tb05870.x 8491209 PMC413443

[48] Mansouri M, Douglas J, Rose PP, et al. Kaposi sarcoma herpesvirus K5 removes CD31/PECAM from endothelial cells. Blood. 2006 Sep 15;108(6):1932–1940. doi:10.1182/blood-2005-11-4404 16601245 PMC1635550

[49] Tugizov S, Maidji E, Xiao J, et al. An Acidic Cluster in the Cytosolic Domain of Human Cytomegalovirus Glycoprotein B Is a Signal for Endocytosis from the Plasma Membrane. J Virol. 1999;73:8677–8688. doi:10.1128/JVI.73.10.8677-8688.1999 10482621 PMC112888

[50] Piguet V, Wan L, Borel C, et al. HIV-1 Nef protein binds to the cellular protein PACS-1 to downregulate class I major histocompatibility complexes. Nat Cell Biol. 2000 Mar;2(3):163–167. doi:10.1038/35004038 10707087 PMC1475706

[51] Crump CM, Xiang Y, Thomas L, et al. PACS-1 binding to adaptors is required for acidic cluster motif-mediated protein traffic. EMBO J. 2001 May 1;20(9):2191–2201. doi:10.1093/emboj/20.9.2191 11331585 PMC125242

[52] Wan L, Molloy SS, Thomas L, et al. PACS-1 defines a novel gene family of cytosolic sorting proteins required for trans-Golgi network localization. Cell. 1998 Jul 24;94(2):205–216. doi:10.1016/S0092-8674(00)81420-8 9695949

[53] Kottgen M, Benzing T, Simmen T, et al. Trafficking of TRPP2 by PACS proteins represents a novel mechanism of ion channel regulation. EMBO J. 2005 Feb 23;24(4):705–716. doi:10.1038/sj.emboj.7600566 15692563 PMC549624

[54] Myhill N, Lynes EM, Nanji JA, et al. The subcellular distribution of calnexin is mediated by PACS-2. Mol Biol Cell. 2008 Jul;19(7):2777–2788. doi:10.1091/mbc.e07-10-0995 18417615 PMC2441662

[55] Dombernowsky SL, Samsoe-Petersen J, Petersen CH, et al. The sorting protein PACS-2 promotes ErbB signalling by regulating recycling of the metalloproteinase ADAM17. Nat Commun. 2015 Jun 25;6:7518. doi:10.1038/ncomms8518 26108729 PMC4481878

[56] Scott GK, Fei H, Thomas L, et al. A PACS-1, GGA3 and CK2 complex regulates CI-MPR trafficking. EMBO J. 2006 Oct 4;25(19):4423–4435. doi:10.1038/sj.emboj.7601336 16977309 PMC1589982

[57] Bergeron E, Albarino CG, Khristova ML, et al. Crimean-Congo hemorrhagic fever virus-encoded ovarian tumor protease activity is dispensable for virus RNA polymerase function. J Virol. 2010 Jan;84(1):216–226. doi:10.1128/JVI.01859-09 19864393 PMC2798392

[58] Suda Y, Fukushi S, Tani H, et al. Analysis of the entry mechanism of Crimean-Congo hemorrhagic fever virus, using a vesicular stomatitis virus pseudotyping system. Arch Virol. 2016 Jun;161(6):1447–1454. doi:10.1007/s00705-016-2803-1 26935918 PMC7087235

[59] Peyrefitte CN, Perret M, Garcia S, et al. Differential activation profiles of Crimean-Congo hemorrhagic fever virus- and Dugbe virus-infected antigen-presenting cells. J Gen Virol. 2010 Jan;91(Pt 1):189–198. doi:10.1099/vir.0.015701-0 19812268

[60] Boson B, Mialon C, Schichl K, et al. Nup98 Is Subverted from Annulate Lamellae by Hepatitis C Virus Core Protein to Foster Viral Assembly. mBio. 2022 Apr 26;13(2):e0292321. doi:10.1128/mbio.02923-21 35258330 PMC9040885

