# Supplementary material for: The PACS-2 protein and trafficking motifs in CCHFV Gn and Gc cytoplasmic domains govern CCHFV assembly
Source: Emerg Microbes Infect. 2024 Apr 25;13(1):2348508. doi: 10.1080/22221751.2024.2348508 (PMC11159592; doi:10.1080/22221751.2024.2348508)
Supplement: Supplementary_Material_Hot_winters_precede_more_intense_WNV_clean [file TEMI_A_2348508_SM6629.docx]

**Warm winters are associated to more intense West Nile virus circulation in southern Spain**

**Sergio Magallanes^1,2^, Francisco Llorente^3^, María José Ruiz-López^1,2^, Josué Martínez-de la Puente^4,2^, Martina Ferraguti^1,2^, Rafael Gutiérrez-López^5,6^, Ramón Soriguer^1,2^, Pilar Aguilera-Sepúlveda ^3^, Raúl Fernández-Delgado^3^, Miguel Ángel Jímenez-Clavero^3,2^, Jordi Figuerola^1,2^**

*1* *Department of Conservation Biology and Global Change, Estación Biológica de Doñana (EBD), CSIC, Seville, Spain.*

*2 CIBER of Epidemiology and Public Health (CIBERESP), Spain.*

*3 Centro de Investigación en Sanidad Animal (CISA-INIA), CSIC, 28130 Valdeolmos, Madrid, Spain.*

*4 Department of Parasitology, University of Granada, Granada E-18071, Spain.*

*5 National Center for Microbiology, Instituto de Salud Carlos III (CNM-ISCIII), 28220, Majadahonda, Madrid, Spain.*

*6 CIBER of Infectious Diseases (CIBERINFEC), Spain*

Correspondence to SMA:

Tel: (+34) 954 232 340

E-mail: sergio.magallanes@ebd.csic.es

Running headline: Eurasian Coot and long-Term surveillance of WNV

S. Magallanes et al.:

**This file includes:**

**Supplementary Table 1.** Description of the climatic variables analysed.

| Variable name | Description of variable | |
| --- | --- | --- |
| *Mean Maximum Winter Temperature* | | Monthly mean of the maximum temperature recorded daily in winter (Cº). |
| *Mean Maximum Spring Temperature* | | Monthly mean of the maximum temperature recorded daily in spring (Cº). |
| *Mean Maximum Summer Temperature* | | Monthly mean of the maximum temperature recorded daily in summer (Cº). |
| *Mean Maximum Autumn Temperature* | | Monthly mean of the maximum temperature recorded daily in autumn (Cº). |
| *Mean Winter Temperature* | | Monthly mean of the temperature recorded daily in winter (Cº). |
| *Mean Spring Temperature* | | Monthly mean of the temperature recorded daily in spring (Cº). |
| *Mean Summer Temperature* | | Monthly mean of the temperature recorded daily in summer (Cº). |
| *Mean Autumn Temperature* | | Monthly mean of the temperature recorded daily in autumn (Cº). |
| *Mean Minimum Winter Temperature* | | Monthly mean of the minimum temperature recorded daily in winter (Cº). |
| *Mean Minimum Spring Temperature* | | Monthly mean of the minimum temperature recorded daily in spring (Cº). |
| *Mean Minimum Summer Temperature* | | Monthly mean of the minimum temperature recorded daily in summer (Cº). |
| *Mean Minimum Autumn Temperature* | | Monthly mean of the minimum temperature recorded daily in autumn (Cº). |
| *Annual Mean Maximum Temperature* | | Annually mean of the maximum temperature recorded daily (Cº). |
| *Annual Mean Temperature* | | Annually mean of the temperature recorded daily (Cº). |
| *Annual Mean Minimum Temperature* | | Annually mean of the minimum temperature recorded daily (Cº). |
| *Winter Mean Rainfall* | | Accumulated rainfall over the whole *Winter* (mm). |
| *Spring Mean Rainfall* | | Accumulated rainfall over the whole *Spring* (mm). |
| *Summer Mean Rainfall* | | Accumulated rainfall over the whole *Summer* (mm/day). |
| *Autumn Mean Rainfall* | | Accumulated rainfall over the whole *Autumn* (mm/day). |
| *Annual Mean Rainfall* | | Accumulated rainfall over the whole year (mm). |

**Supplementary Table 2.** Prevalence of WNV antibodies detected in all the common coots samples was estimated from the back-transformed *LSMeans* estimates to account for the effects of the variables included in the statistical model.

| Variable | Level | Prevalence (%) | S.E. | Sample size |
| --- | --- | --- | --- | --- |
| **Season** | Autumn | 22.3 | 0.04 | 1647 |
|  | Winter | 33.3 | 0.06 | 546 |
|  | Spring | 15.6 | 0.04 | 78 |
|  | Summer | 13.9 | 0.03 | 453 |
| **Age group** | Juveniles | 17.6 | 0.04 | 626 |
|  | Adults | 23.5 | 0.05 | 2098 |

**Supplementary Table 3.** Results of the GLMM analysing relationships between the seroprevalence of WNV antibodies with age category (adult and young), and season of sampling (autumn, winter, spring and summer) (N = 2,724).

| VARIABLES | CATEGORY | ESTIMATE | *S.E.* | *z* | *p* | Post Hoc |
| --- | --- | --- | --- | --- | --- | --- |
| *INTERCEPT* |  | -1.067 | 0.243 | -4.40 | <0.001* |  |
| *Season* | Autumn | 0^(a)^ |  |  |  | A |
|  | Winter | 0.553 | 0.144 | 3.83 | <0.001*** | C |
|  | Spring | -0.439 | 0.331 | -1.33 | 0.185 | BA |
|  | Summer | -0.574 | 0.176 | -3.26 | 0.001** | B |
| *Age Class* | Adult | 0^(a)^ |  |  |  | A |
|  | Young | -0.360 | 0.146 | -2.46 | 0.014* | B |

**Supplementary Table 4.** Results of the GLMM analysing the rest of the climate variables discarded after forward stepwise selection to construct the best regression model. These variables are included one by one in the final model.

| Variables | Z | P |
| --- | --- | --- |
| **Mean Maximum Spring Temperature** | -0.434 | 0.664 |
| **Mean Maximum Summer Temperature** | -1.310 | 0.190 |
| **Mean Maximum Autumn Temperature** | -0.005 | 0.995 |
| **Mean Winter Temperature** | -0.249 | 0.803 |
| **Mean Summer Temperature** | -0.209 | 0.834 |
| **Mean Autumn Temperature** | -1.310 | 0.190 |
| **Mean Minimum Winter Temperature** | -0.636 | 0.524 |
| **Mean Minimum Spring Temperature** | 0.299 | 0.765 |
| **Mean Minimum Summer Temperature** | -0.605 | 0.544 |
| **Mean Minimum Autumn Temperature** | -0.262 | 0.793 |
| **Annual Maximum Temperature** | -0.502 | 0.615 |
| **Annual Mean Temperature** | -0.617 | 0.537 |
| **Annual Mean Minimum Temperature** | -0.593 | 0.553 |
| **Winter Mean Rainfall** | -1.429 | 0.152 |
| **Spring Mean Rainfall** | 0.362 | 0.717 |
| **Summer Mean Rainfall** | -0.584 | 0.559 |
| **Autumn Mean Rainfall** | 0.721 | 0.470 |
| **Annual Mean Rainfall** | 0.031 | 0.975 |
